# Implementation of a National Reference Laboratory for Buruli Ulcer Disease in Togo

**DOI:** 10.1371/journal.pntd.0002011

**Published:** 2013-01-24

**Authors:** Marcus Beissner, Kristina Lydia Huber, Kossi Badziklou, Wemboo Afiwa Halatoko, Issaka Maman, Felix Vogel, Bawimodom Bidjada, Koffi Somenou Awoussi, Ebekalisai Piten, Kerstin Helfrich, Carolin Mengele, Jörg Nitschke, Komi Amekuse, Franz Xaver Wiedemann, Adolf Diefenhardt, Basile Kobara, Karl–Heinz Herbinger, Abiba Banla Kere, Mireille Prince-David, Thomas Löscher, Gisela Bretzel

**Affiliations:** 1 Department of Infectious Diseases and Tropical Medicine (DITM), University Hospital, Ludwig-Maximilians-University, Munich, Germany; 2 Institut National d'Hygiène (INH), Ministry of Health, Lomé, Togo; 3 Institute for Applied Informatics and Formal Description Methods, Karlsruhe Institute of Technology (KIT), Karlsruhe, Germany; 4 Centre Hospitalier Régional Maritime (CHR Maritime), Tsévié, Togo; 5 German Leprosy and Tuberculosis Relief Association (DAHW), Würzburg, Germany; 6 German Leprosy and Tuberculosis Relief Association, Togo Office (DAHWT), Lomé, Togo; 7 Programme National de Lutte contre l'Ulcère de Buruli – Lèpre et Pian (PNLUB-LP), Ministry of Health, Lomé, Togo; 8 Département des Sciences Fondamentales et Biologiques, Faculté Mixte de Médecine et de Pharmacie (FMMP), Université de Lomé (UL), Lomé, Togo; Fondation Raoul Follereau, France

## Abstract

**Background:**

In a previous study PCR analysis of clinical samples from suspected cases of Buruli ulcer disease (BUD) from Togo and external quality assurance (EQA) for local microscopy were conducted at an external reference laboratory in Germany. The relatively poor performance of local microscopy as well as effort and time associated with shipment of PCR samples necessitated the implementation of stringent EQA measures and availability of local laboratory capacity. This study describes the approach to implementation of a national BUD reference laboratory in Togo.

**Methodology:**

Large scale outreach activities accompanied by regular training programs for health care professionals were conducted in the regions “Maritime” and “Central,” standard operating procedures defined all processes in participating laboratories (regional, national and external reference laboratories) as well as the interaction between laboratories and partners in the field. Microscopy was conducted at regional level and slides were subjected to EQA at national and external reference laboratories. For PCR analysis, sample pairs were collected and subjected to a dry-reagent-based IS*2404*-PCR (DRB-PCR) at national level and standard IS*2404* PCR followed by IS*2404* qPCR analysis of negative samples at the external reference laboratory.

**Principal Findings:**

The inter-laboratory concordance rates for microscopy ranged from 89% to 94%; overall, microscopy confirmed 50% of all suspected BUD cases. The inter-laboratory concordance rate for PCR was 96% with an overall PCR case confirmation rate of 78%. Compared to a previous study, the rate of BUD patients with non-ulcerative lesions increased from 37% to 50%, the mean duration of disease before clinical diagnosis decreased significantly from 182.6 to 82.1 days among patients with ulcerative lesions, and the percentage of category III lesions decreased from 30.3% to 19.2%.

**Conclusions:**

High inter-laboratory concordance rates as well as case confirmation rates of 50% (microscopy), 71% (PCR at national level), and 78% (including qPCR confirmation at external reference laboratory) suggest high standards of BUD diagnostics. The increase of non-ulcerative lesions, as well as the decrease in diagnostic delay and category III lesions, prove the effect of comprehensive EQA and training measures involving also procedures outside the laboratory.

## Author Summary

Buruli ulcer disease (BUD), the third most common mycobacterial disease worldwide, is treated with standardized antimycobacterial therapy. According to WHO recommendations at least 50% of cases should be laboratory confirmed by polymerase chain reaction (PCR). In a previous study PCR analysis of clinical samples from suspected BUD cases from Togo and external quality assurance (EQA) for local microscopy were conducted at an external reference laboratory in Germany. The relatively poor performance of local microscopy as well as time and effort associated with shipment of clinical samples abroad necessitated the availability of a local BUD reference laboratory and the implementation of stringent EQA measures. All processes in the laboratories as well as in the field were defined by standard operating procedures, microscopy conducted at regional facilities was subjected to EQA at national and external reference level, and PCR samples were analyzed in parallel at national and external reference laboratories. Inter-laboratory concordance rates of >90% and case confirmation rates of 50% (microscopy) and >70% (PCR) respectively suggest high standards of BUD diagnostics. Furthermore, an increase of non-ulcerative lesions and a decrease in diagnostic delay and category III lesions reflect the impact of comprehensive EQA measures also involving procedures outside the laboratory on the quality of BUD control.

## Introduction

Buruli ulcer disease (BUD), caused by *Mycobacterium ulcerans*, is an infectious disease affecting skin, soft tissue and bones. If left untreated, extensive destruction of tissue followed by fibrous scarring and contractures may lead to severe functional limitations [1]–[6]. BUD is treated with rifampicin and streptomycin (or clarithromycin) for eight weeks if necessary followed by surgical interventions; the laboratory confirmation of clinically suspected BUD cases prior to treatment has become an integral part of clinical management. Whereas microscopy is an appropriate and cost-effective first-line test for peripheral laboratories, PCR is considered the method of choice and WHO recommends PCR confirmation of at least 50% of suspected BUD cases [3], [7]–[13]. Microscopy and various PCR assays have been successfully implemented in other endemic countries and case confirmation rates of 29–78% (microscopy) and 54–83% (PCR) were reported [10], [12]–[32].

Since the early 1990s, close to 2,000 BUD cases were reported from Togo. However, due to the lack of local diagnostic laboratory capacity, the majority of these cases remained unconfirmed [7], [13], [33]–[35].

From 2007 through 2010, a joint research project between the German Leprosy and Tuberculosis Relief Organization, Togo office, Lomé, Togo (DAHWT) and the Department of Infectious Diseases and Tropical Medicine (DITM), University Hospital, Ludwig-Maximilians-University, Munich, Germany, allowed the first systematic study on laboratory confirmation of BUD cases from Togo and proved the prevalence of BUD in South Togo (region “Maritime”). The study revealed a relatively poor performance of local Ziehl-Neelsen microscopy, suggesting the need for a stringent system for external quality assurance (EQA) including regular supervision of microscopy laboratories. Intensified training measures in the area of sample collection resulted in a PCR case confirmation rate of 70%. Effort and turnaround time associated with shipment of samples to an external reference laboratory, however, necessitated the availability of local laboratory capacities [13].

In the context of the EC-funded research project “BuruliVac” (FP7/2010–2013; grant agreement N° 241500), the implementation of a national reference laboratory for BUD in Togo was envisaged. Therefore, from January 2011 through April 2012, microscopy and PCR facilities were established at the “Institut National d'Hygiène” (INH), Lomé, Togo.

This study describes the approach to implementation of a national reference laboratory and analyzes the impact of intensified EQA and training measures on laboratory diagnosis and control of BUD in Togo.

## Materials and Methods

### Ethics

Ethical clearance was obtained through the national Togolese ethics committee (“Comité de Bioéthique pour la Recherche en Santé”) at the University of Lomé (14/2010/CBRS) and the study was approved by the “Ministère de la Santé de la République Togolaise” Lomé, Togo (Ref. No. 0009/2011/MS/DGS/DPLET). All samples analyzed in this study were collected for diagnostic purposes within the EC funded research project “BuruliVac”. Written informed consent was obtained from all study participants.

### Role of participating institutions

This study constitutes a collaborative project between several Togolese and German institutions. Since 2007, the German Leprosy and Tuberculosis Relief Organization (DAHW) has supported the Togolese National Buruli Ulcer Control Program (“Programme National de Lutte contre L'Ulcère de Buruli – Lèpre et Pian” [PNLUB-LP]) in the area of training, laboratory confirmation and treatment of BUD. In this study, the main tasks of DAHWT, as partner of the “BuruliVac” consortium were field work, recruitment of study participants, and collection of diagnostic samples. The tasks of DITM – an accredited laboratory according to DIN EN ISO 15189 - as lead partner for all patient related activities of the “BuruliVac” project consisted of implementation of molecular diagnostic laboratory methods at the designated national Togolese BUD reference laboratory and standardization of all processes through on-site training, standard operating procedures (SOPs), and EQA of microscopy and PCR (by standard gel-based IS*2404* PCR and IS*2404* quantitative real-time PCR [qPCR]) including supervisory visits. Patients with suspected BUD were referred to peripheral health posts (“Unité de Soins Périphérique”, USP; operating on district level as point of care facilities with a catchment area of 5,000–9,000 inhabitants depending on the number of facilities per district), or a regional hospital (“Centre Hospitalier Régional [CHR] de Tsévié”, region “Maritime”, Togo, since 2007 national reference centre for BUD in Togo; catchment area: 2,599,955 inhabitants) for diagnosis and treatment; CHR conducted microscopic analysis. The “Institut National d'Hygiène” (INH), Lomé, Togo – a laboratory accredited by COFRAC (“Comité Français d'Accréditation”) according to NF EN ISO/CEI 17025 (version 2005) – constitutes the national Togolese reference laboratory for surveillance of transmissible, especially outbreak prone diseases, and has been nominated national reference laboratory for Buruli ulcer disease in 2010 [13]. In this study, INH resumed EQA for microscopy conducted at regional level and – after installation of a BUD PCR laboratory – PCR assessment of diagnostic samples by means of a dry-reagent-based PCR [21], [25], [29]. In March 2011, INH joined the WHO network for laboratory confirmation of BUD and – like DITM – participates in the annual program for external quality assessment of molecular detection of *M. ulcerans* in clinical specimens provided by the Mycobacteriology Unit, Microbiology Department, Institute for Tropical Medicine, Antwerp, Belgium, WHO Collaborating Centre for the diagnosis and surveillance of *M. ulcerans* infection [36].

### Study area and implementation of outreach programs

In each of the six districts (Golfe, Ave, Zio, Yoto, Vo, Lac) of the region “Maritime”, five districts (“Direction de District Sanitaire” [DDS] 1–5) of the region “Lomé Commune” where BUD was proven to be endemic [13] and the four districts of the region “Central” (Blitta, Sotouboua, Tchaoudjo, Thamba), where BUD has been assumed to be endemic, outreach teams (“CLT teams”) consisting of district controllers (“Contrôleur Lèpre-TB-Buruli”, CLT), USP staff (“Infirmière du Centre Peripherique”, ICP) and community health workers (“Agent de Santé Communautaire”, ASC), and village nurses were formed and trained by experienced PNLUB-LP, CHR, DITM and DAHW staff. The main tasks of the CLT teams are supervision of USPs, as well as sensitization and screening activities in the field which are mostly conducted under participation of DAHW and CHR staff and in collaboration with PNLUB-LP and the non-governmental organization Handicap International. In particular the ASCs who are trained and continuously supervised by the respective CLTs constitute an integral part of the outreach activities. They organize quarterly sensitization activities and present educational films and information material in villages within proven or assumed areas of endemicity. Villagers are instructed to report to their local ASCs in case of wounds or other lesions suspicious for BUD, thus ASCs represent the primary contact person for the population on community level. Furthermore, ASCs organize regular screening programs in village schools to identify suspected BUD cases in the field. The final decision on referral of suspected BUD cases to USPs or CHR for further diagnosis and treatment lies with a superordinate “BUD team” consisting of medical staff (physician, nurse) from CHR, ASCs, and the regional CLT. Visits to field sites are conducted on demand of district CLT teams according to a schedule elaborated by the ASCs. A routine reporting system between ASCs, ICPs, CLTs and CHR staff has been established and to facilitate communication within and between CLT teams and BUD teams a mobile phone network has been implemented by DAHW in 2010.

### Data management

Data collection was conducted by means of the WHO “BU01” form [3] and standardized project specific laboratory data entry forms (Form S1). All clinical, epidemiological and laboratory data including EQA results were entered in a web-based database specifically designed for the “BuruliVac” project.

### Sample collection

Diagnostic samples were collected according to standardized procedures. Briefly, swabs were collected by circling the entire undermined edges of ulcerative lesions. Three millimeter punch biopsies and fine needle aspirates (FNA) were collected from the center of non-ulcerative lesions or from undermined edges of ulcerative lesions including necrotic tissue. To facilitate sampling, standardized specimen collection bags including swabs, biopsy punches, syringes and needles, slides, containers with transport media (700 µl [swab and punch biopsy samples], 300 µl [FNA samples] CLS [cell lysis solution, Qiagen, Hilden, Germany] for PCR samples) and data entry forms were provided to the study sites [13], [23], [25], [26], [29], [37]–[41].

Samples for PCR analysis were transported in CLS at ambient temperature in an upright position in custom-made specimen collection bags from the field to INH by DAHWT cars within a maximum of 48 hours following sample collection. Upon arrival of PCR samples at INH these were stored at 4–8°C until further processing. Slides for microscopy were transported in slide boxes at ambient temperature to CHR and subsequently to INH.

### Laboratory diagnostics

Direct smears for microscopy were prepared from swab and FNA samples at USPs or CHR and subjected to Ziehl-Neelsen staining at CHR. Slides were analyzed according to the WHO recommended grading system [42].

For PCR analysis DNA was prepared using the Gentra Puregene DNA extraction kit (Qiagen, Hilden, Germany) with minor modifications of the manufacturer's protocol [21].

Three IS*2404* PCR formats (dry-reagent-based [DRB] IS*2404* PCR [INH], standard gel-based IS*2404* PCR and IS*2404* qPCR [DITM]) were applied in this study. Briefly, for DRB-PCR the oligonucleotides MU5 and MU6 were lyophilized in reaction tubes. Illustra PuReTaq Ready-To-Go PCR beads (GE Healthcare, Munich, Germany) were added and dissolved in water before adding template DNA [21], [25], [26]. Standard IS*2404* PCR was performed according to the protocol described by Stinear et al. [15], [17]. IS*2404* qPCR was performed as recently described using a BioRad CFX96 real-time PCR detection system [27], [43]. All PCR assays included negative extraction controls, positive, negative (no template) and inhibition controls.

### Stepwise approach to implementation of diagnostic laboratory facilities at INH

**Figure 1:**
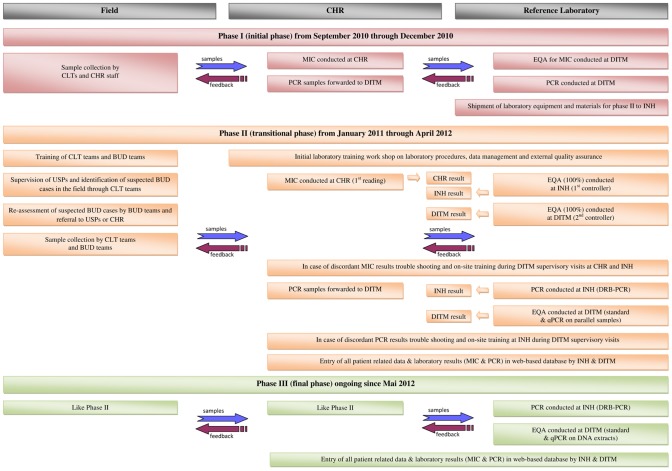
Stepwise approach to implementation of diagnostic laboratory facilities at INH. Figure 1 describes the process of implementation of diagnostic laboratory facilities at INH in three phases and the flow of samples as well as the flow of feedback between the Department for Infectious Diseases and Tropical Medicine (DITM), Ludwig-Maximilians-University, Munich, Germany, the “Institut National d'Hygiène” (INH), Lomé, Togo, the “Centre Hospitalier Régional Maritime” (CHR), Tsévié, Togo, and field staff. BUD, Buruli ulcer disease; CLT, “Contrôleur Lèpre-TB-Buruli” – district controllers; DRB-PCR, dry-reagent-based IS*2404* PCR; EQA, external quality assurance; MIC, microscopic detection of acid fast bacilli by Ziehl-Neelsen staining; PCR, polymerase chain reaction; qPCR, IS*2404* quantitative real-time PCR; standard PCR, conventional gel-based IS*2404* PCR; USP, “Unité de Soins Périphérique” – peripheral health posts.

Implementation of diagnostic laboratory facilities at INH was accomplished in several phases. Before launching the national BUD reference laboratory at INH in January 2011, laboratory assessment of diagnostic samples from “BuruliVac” study participants was conducted at CHR (microscopy) and DITM (PCR) respectively (“initial phase” [phase I] from September 2010 through December 2010). To implement standardized BUD microscopy and PCR services at INH, all required equipment, reagents and consumables were shipped to Togo by DAHWT and installed under supervision of DITM staff from November through December 2010. Subsequently, the transitional phase (phase II) was initiated in January 2011. All relevant laboratory procedures were defined in SOPs (SOP S1–S4). An initial laboratory training workshop was held by DITM staff, and INH staff was familiarized with the principles of standardized documentation of samples and corresponding results (laboratory data entry forms, web-based database), the flow of information between the participating laboratories, and the principles of EQA as outlined below. Whereas during the transitional phase from January 2011 through April 2012 parallel diagnostic samples of all study participants were simultaneously subjected to PCR analysis at INH and DITM, the final phase (phase III) of PCR implementation (ongoing since May 2012) provides for diagnostic PCR conducted independently at INH accompanied by EQA on DNA extracts at DITM. (Figure 1)

### External Quality Assurance

During the initial phase EQA was conducted for microscopy only. Slides were read at CHR by two readers, forwarded to DITM for blinded re-reading [13], and both, CHR and DITM results were entered in the web-based database. In case of discordant results between CHR and DITM, slides were subjected to a second re-reading at DITM which determined the consensus result.

During the transitional phase CHR conducted the first reading of slides by two readers, entered a consensus result in a specific result form (Form S1), and forwarded slides and forms to INH (first controller) for blinded re-reading. INH consensus results were also determined by two readers and entered in a specific result form (Forms S2). Finally, CHR and INH results were entered in the web-based database by INH data managers. In case of discordant results the respective slides were re-read by both, CHR and INH staff, and a consensus result was determined. Subsequently, slides were forwarded to DITM (second controller) for blinded re-reading, and DITM results were entered in the web-based database. Slides with discordant results between DITM and INH were re-read by DITM and INH staff during DITM supervisory visits.

For EQA of PCR all clinical samples were collected in pairs and were simultaneously tested at INH (DRB-PCR) and DITM (standard IS*2404* PCR, confirmatory IS*2404* qPCR on negative samples). Results were entered in the web-based database. In case of discordant results both laboratories repeated PCR analyses. If the result did not alter, DNA extracts of the respective samples were exchanged and re-tested at both laboratories.

### Parameters to determine performance of CHR and INH

In accordance with a previous study on EQA for the laboratory diagnosis of BUD in Ghana [23] microscopy positivity rates (i.e. number of positive samples divided by the total number of samples tested) at CHR, INH, and DITM, PCR positivity rates at INH and DITM, rates of false negative and false positive results compared to DITM results and inter-laboratory concordance rates between CHR/INH/DITM for microscopy and INH/DITM for PCR were determined for the initial and transitional phases. In addition, case confirmation rates (i.e. number of laboratory confirmed BUD patients divided by the total number of suspected BUD cases) were determined for CHR (microscopy), INH and DITM (microscopy and PCR).

### Parameters to assess the impact on BUD control

To assess the impact of the local reference laboratory and continuous EQA measures on BUD control, the clinical parameters “type of lesion”, “category of lesion”, and “duration of disease before clinical diagnosis” (i.e. the mean duration of disease in days based on the time from first recognition of clinical symptoms by patients and availability of the clinical diagnosis BUD) were analyzed and data obtained from the current study cohort from January 2011 through April 2012 after implementation of the national reference laboratory were compared to data obtained in a previous study from September 2007 through December 2010.

### Feedback to CHR and field staff

INH forwards all laboratory results directly to CHR, the subsequent reporting chain includes regional CLTs, district CLTs, ICPs, and ASCs. Laboratory confirmed BUD patients are subjected to treatment. In case of negative laboratory results in general the treatment decision is referred to the BUD team. For the purpose of documentation, lesions of all confirmed patients are photographed; the material is available for training and sensitization activities.

### Statistical analysis

The study design was non-randomized and cross-sectional.

Approximative tests (χ^2^-tests) including analysis for linear trends in proportions and t-tests as parametric test were conducted using Stata software, version 9.0. (Stata Corporation, College Station, TX) and EpiInfo, version 3.3.2. (Centers for Disease Control and Prevention, Atlanta, GA). Significant differences were defined as not overlapping of 95 percent confidence intervals (95% CI) of proportions.

## Results

### Training measures for field staff and outcome of outreach programs

Altogether 16 workshops with 559 participants (“CLT teams” as well as other medical and paramedical staff) addressing clinical picture, laboratory diagnosis and treatment of BUD were held in the regions “Maritime” and “Central”. Since 2011, the CLT teams conducted sensitization activities in 1027 villages and screened a population of approximately 110,000. Out of 192 persons with lesions suspicious for BUD identified in the field, 82 suspected BUD cases were finally referred to USPs or CHR. (Table 1)

**Table 1:** Geographic origin, type and classification of lesions of clinically suspected and laboratory confirmed BUD patients.

Region	District	Clinically suspected BUD cases	Laboratory confirmed BUD patients^a^					
			Total per district^b^	Non-ulcerative lesions	Ulcerative lesions	Category I^c^	Category II^d^	Category III^e^
Central	Sotouboua	4.9% (4/82)	0% (0/64)	0% (0/64)	0% (0/64)	0% (0/64)	0% (0/64)	0% (0/64)
Maritime	Golfe	2.4% (2/82)	1.6% (1/64)	0% (0/64)	1.6% (1/64)	0% (0/64)	0% (0/64)	1.6% (1/64)
	Yoto	48.8% (40/82)	57.8% (37/64)	31.3% (20/64)	26.6% (17/64)	32.8% (21/64)	18.8% (12/64)	6.3% (4/64)
	Vo	1.2% (1/82)	1.6% (1/64)	1.6% (1/64)	0% (0/64)	1.6% (1/64)	0% (0/64)	0% (0/64)
	Zio	36.6% (30/82)	34.4% (22/64)	18.8% (12/64)	15.6% (10/64)	9.4% (6/64)	18.8% (12/64)	6.3% (4/64)
Plateaux^f^	Amoú	1.2% (1/82)	0% (0/64)	0% (0/64)	0% (0/64)	0% (0/64)	0% (0/64)	0% (0/64)
	Anié	1.2% (1/82)	1.6% (1/64)	0% (0/64)	1.6% (1/64)	0% (0/64)	1.6% (1/64)	0% (0/64)
	Haho	1.2% (1/82)	0% (0/64)	0% (0/64)	0% (0/64)	0% (0/64)	0% (0/64)	0% (0/64)
	Ogou	1.2% (1/82)	1.6% (1/64)	0% (0/64)	1.6% (1/64)	0% (0/64)	0% (0/64)	1.6% (1/64)
Savanes^f^	Dapaong	1.2% (1/82)	1.6% (1/64)	0% (0/64)	1.6% (1/64)	0% (0/64)	1.6% (1/64)	0% (0/64)
**Total**		**100% (82/82)**	**100% (64/64)**	**51.6% (33/64)**	**48.4% (31/64)**	**43.8% (28/64)**	**40.6% (26/64)**	**15.6% (10/64)**

Table 1 shows the geographic origin of all suspected and confirmed BUD patients, and type/category of lesions of confirmed BUD patients who presented from September 2010 through April 2012 in Togo. More than 85% of confirmed BUD patients originated from the districts Yoto and Zio of region “Maritime”.

a Patients were confirmed by dry-reagent-based IS*2404*, standard gel-based IS*2404* PCR and/or IS*2404* quantitative real-time PCR. BUD, Buruli ulcer disease.

b Number of confirmed BUD patients per district.

c Category I, single lesion <50 mm in diameter.

d Category II, single lesion between 50 and 150 mm in diameter.

e Category III, single lesion >150 mm in diameter or multiple lesions, osteomyelitis or lesions at critical sites.

f Laboratory confirmed BUD patients were referred to CHR, Tsévié, for antimycobacterial treatment.

### Number of samples analyzed by microscopy

During the initial phase, 17 slides (swab, n = 6; FNA, n = 11) obtained from 16 suspected BUD cases (ten non-ulcerative lesions: one FNA sample per lesion; six ulcerative lesions, one swab sample per lesion and one additional FNA sample from one lesion with scarred edges) were analyzed at CHR and subjected to EQA at DITM.

During the transitional phase, 72 slides (swab, n = 24; FNA, n = 48) obtained from 66 suspected BUD cases (38 non-ulcerative lesions: one FNA sample per lesion; 28 ulcerative lesions: one swab sample each from 18 lesions, one swab and one FNA sample each from six lesions, one FNA sample each from four lesions) were analyzed at CHR and subjected to EQA at INH and DITM. (Table 2)

**Table 2:** Clinical samples analyzed by microscopy for *M. ulcerans*.

	No. of suspected BUD cases	No. of swab samples subjected to MIC^a^		No. of FNA samples subjected to MIC^a^		Total^b^
		CHR/DITM	CHR/INH/DITM	CHR/DITM	CHR/INH/DITM	
Phase I^c^	16	6	N/A	11	N/A	17
Phase II^d^	66	N/A	24	N/A	48	72
Total	82	30		59		89

Table 2 indicates all slides prepared from swab or FNA samples and subjected to Ziehl-Neelsen staining at “Centre Hospitalier Régional” (CHR) for the detection of acid fast bacilli. Slides were analyzed consecutively at CHR and the Department of Infectious Diseases and Tropical Medicine (DITM), Ludwig-Maximilians-University during initial phase (phase I) or CHR, at the “Institut National d'Hygiène” (INH) and DITM during transitional phase (phase II). N/A, not applicable.

a MIC, microscopic detection of acid fast bacilli.

b Total, number of slides prepared from swab and FNA samples and subjected to reading at CHR/DITM or CHR/INH/DITM.

c Phase I, initial phase of implementation of the national reference laboratory at INH from September 2010 through December 2010; slides were read at CHR and forwarded via DAHWT to DITM for EQA.

d Phase II, transitional phase of implementation of the national reference laboratory at INH from January 2011 through April 2012; slides were read at CHR, followed by blinded re-reading at INH and DITM.

### External quality assurance of microscopy

During the initial phase positivity rates of microscopy were 41.2% (7/17) at CHR and 47.1% (8/17) at DITM with 5.9% (1/17) false negative results from CHR, and an inter-laboratory concordance rate of 94.1% (16/17) between CHR and DITM.

During the transitional phase positivity rates of microscopy were 47.2% (34/72) at CHR, 48.6% (35/72) at INH and 55.6% (40/72) at DITM. The rate of false negative test results was 9.7% (7/72) at CHR and 6.9% (5/72) at INH, and 1 out of 72 slides (1.4%) was read false positive at CHR. Concordance rates between laboratories were 94.4% (68/72) for CHR/INH, 88.9% (64/72) for CHR/DITM and 93.1% (67/72) for INH/DITM.

The concordance rate between CHR and DITM for both phases was 89.9% (80/89). (Table 3)

**Table 3:** Results of external quality assurance for microscopy.

	CHR			INH			DITM		
	Swab^a^	FNA^b^	Total	Swab^a^	FNA^b^	Total	Swab^a^	FNA^b^	Total
**Phase I**									
**Positivity rate** c	50.0% (3/6)	36.4% (4/11)	41.2% (7/17)	N/A	N/A	N/A	66.7% (4/6)	36.4% (4/11)	47.1% (8/17)
**False negative results** d	16.7% (1/6)	0% (0/11)	5.9% (1/17)	N/A	N/A	N/A	N/A	N/A	N/A
**False positive results** e	0% (0/6)	0% (0/11)	0% (0/17)	N/A	N/A	N/A	N/A	N/A	N/A
**Discordance rate** f	16.7% (1/6)	0% (0/11)	5.9% (1/17)	N/A	N/A	N/A	N/A	N/A	N/A
**Concordance rate for CHR/DITM** g	83.3% (5/6)	100% (11/11)	94.1% (16/17)	N/A	N/A	N/A	N/A	N/A	N/A
**Case confirmation rate** h	31.3% (5/16)			N/A			37.5% (6/16)		
**Phase II**									
**Positivity rate** c	50.0% (12/24)	45.8% (22/48)	47.2% (34/72)	50.0 (12/24)	47.9% (23/48)	48.6% (35/72)	58.3% (14/24)	54.2% (26/48)	55.6% (40/72)
**False negative results** d	8.3% (2/24)	10.4% (5/48)	9.7% (7/72)	8.3% (2/24)	6.3% (3/48)	6.9% (5/72)	N/A	N/A	N/A
**False positive results** e	0% (0/24)	2.1% (1/48)	1.4% (1/72)	0% (0/24)	0% (0/48)	0% (0/72)	N/A	N/A	N/A
**Discordance rate** f	8.3% (2/24)	12.5% (6/48)	11.1% (8/72)	8.3% (2/24)	6.3% (3/48)	6.9% (5/72)	N/A	N/A	N/A
**Concordance rate for CHR/INH** g	100% (24/24)	91.7% (44/48)	94.4% (68/72)	N/A	N/A	N/A	N/A	N/A	N/A
**Concordance rate for CHR/DITM** g	91.7% (22/24)	87.5% (42/48)	88.9% (64/72)	N/A	N/A	N/A	N/A	N/A	N/A
**Concordance rate for INH/DITM** g	N/A	N/A	N/A	91.7% (22/24)	93.7% (45/48)	93.1% (67/72)	N/A	N/A	N/A
**Case confirmation rate** h	43.9% (29/66)			47.0% (31/66)			53.0% (35/66)		
**Concordance rate** g **for CHR/DITM – Phase I and II**	89.9% (80/89)			N/A			N/A		
**Case confirmation rate** h **– Phase I and II**	41.5% (34/82)			N/A			50.0% (41/82)		

Table 3 shows results of Ziehl-Neelsen microscopy conducted at CHR and corresponding results of EQA conducted at INH and DITM during the initial (phase I, September 2010 through December 2010) and transitional (phase II, January 2011 through April 2012) phases. N/A, not applicable.

a Swab, slides were prepared as direct smears from swab samples.

b FNA, slides were prepared as direct smears from fine-needle aspirate samples.

c Positivity rate, number of positive samples divided by the total number of samples tested.

d Rate of false negative results at CHR and INH, respectively, compared to DITM results.

e Rate of false positive results at CHR and INH, respectively, compared to DITM results.

f Rate of false negative and false positive results at CHR and INH, respectively, compared to DITM results.

g Rate of concordant results between CHR/INH, CHR/DITM and INH/DITM.

h Confirmation rate, number of laboratory confirmed BUD patients divided by the total number of suspected BUD cases.

### Number of samples analyzed by PCR

During the initial phase, 35 samples (swab, n = 6; FNA, n = 16; punch biopsy, n = 13) obtained from 16 suspected BUD cases were subjected to standard PCR at DITM, all negative samples (n = 12) were additionally subjected to qPCR.

During the transitional phase, 99 sample pairs (swab, n = 33; FNA, n = 44; punch biopsy, n = 22) obtained from 66 suspected BUD cases were subjected to PCR at INH and DITM, which equals a mean rate of 3.0 (198/66) samples tested per patient. All negative samples (n = 30) were additionally subjected to qPCR. (Table 4)

**Table 4:** Clinical samples analyzed by PCR for *M. ulcerans*.

	No. of suspected BUD cases	Samples analyzed by PCR					
		Laboratory	IS*2404* PCR assay	Swab^a^	FNA^b^	Punch^c^	Total
**Phase I** d	16	DITM	Standard PCR	6	16	13	35
			qPCR^e^	3/6	6/16	3/13	12/35
		**Total** f		6	16	13	35
**Phase II** g	66	INH	DRB PCR	33	44	22	99
		DITM	Standard PCR	33	44	22	99
			qPCR^e^	6/33	15/44	9/22	30/99
		**Total** f		66	88	44	198
**Total -phase I and II**	**82**			**72**	**104**	**57**	**233**

Table 4 indicates all samples tested by PCR at IHN and DITM. During the initial phase (phase I) samples were analyzed by standard gel-based IS*2404* PCR at DITM. During the second phase (phase II) parallel samples were subjected to IS*2404* dry-reagent based (DRB) PCR at INH and standard IS*2404* PCR at DITM. During both phases all samples tested negative in standard PCR were subjected to re-testing by IS*2404* quantitative real-time PCR (qPCR) at DITM.

a Swab, DNA extracts prepared from swab samples.

b FNA, DNA extracts prepared from fine-needle aspirate samples.

c Punch, DNA extracts prepared from 3 mm punch biopsy samples.

d Phase I, initial phase of implementation of the national reference laboratory at INH from September through December 2010.

e Only samples tested negative in standard IS*2404* PCR were subjected to IS*2404* qPCR at DITM.

f Total amount of samples tested by DRB- and Standard PCR during the corresponding phases.

g Phase II, transitional phase of implementation of the national reference laboratory at INH from January 2011 through April 2012.

### External quality assurance of PCR

During the initial phase the positivity rate of standard PCR at DITM was 65.7% (23/35). Confirmation of two out of 12 negative samples by qPCR provided an additional diagnostic yield of 5.7%.

During the transitional phase positivity rates of conventional PCR assays were 65.7% (65/99) at INH and 69.7% (69/99) at DITM. The rate of false negative test results at INH was 4.0% (4/99; 1 swab sample and 3 FNA samples), there were no false positive results, and the inter-laboratory concordance rate was 96.0% (95/99). Confirmation of 6 out of 30 negative samples by qPCR provided an additional diagnostic yield of 6.1%. (Table 5)

**Table 5:** Results of EQA for PCR.

		Swab samples^a^			FNA samples^b^			Punch biopsy samples^c^			Total^d^			
		INH^e^	DITM^f^		INH^e^	DITM^f^		INH^e^	DITM^f^		INH^e^	DITM^f^		
		DRB-PCR^e^	Standard PCR^f^	qPCR^f^	DRB-PCR^e^	Standard PCR^f^	qPCR^f^	DRB-PCR^e^	Standard PCR^f^	qPCR^f^	DRB-PCR^e^	Standard PCR^f^	qPCR^f^	Final result^g^
**Phase I** h	**Positivity rate** i	N/A	50.0% (3/6)	0% (0/3)	N/A	62.5% (10/16)	16.7% (1/6)	N/A	76.9% (10/13)	33.3% (1/3)	N/A	65.7% (23/35)	16.7% (2/12)	71.4% (25/35)
	**Case confirmation rate** j	N/A	18.8% (3/16)	0% (0/16)	N/A	43.8% (7/16)	0% (0/16)	N/A	12.5% (2/16)	0% (0/16)	N/A	75.0% (12/16)	0% (0/16)	75.0% (12/16)
**Phase II** m	**Positivity rate** i	78.8% (26/33)	81.8% (27/33)	16.7% (1/6)	59.1% (26/44)	66.0% (29/44)	33.3% (5/15)	59.1% (13/22)	59.1% (13/22)	0.0% (0/9)	65.7% (65/99)	69.7% (69/99)	20.0% (6/30)	75.8% (75/99)
	**False negative** k	3.0% (1/33)	N/A	N/A	6.8% (3/44)	N/A	N/A	0.0% (0/22)	N/A	N/A	4.0% (4/99)	N/A	N/A	N/A
	**Concordance rate** l	97.0% (32/33)		N/A	93.2% (41/44)		N/A	100% (22/22)		N/A	96.0% (95/99)		N/A	N/A
	**Case confirmation rate** j	30.3% (20/66)	30.3% (20/66)	0.0% (0/66)	34.8% (23/66)	39.4% (26/66)	3.0% (2/66)	6.1% (4/66)	6.1% (4/66)	0.0% (0/66)	71.2% (47/66)	75.8% (50/66)	3.0% (2/66)	78.8% (52/66)
**Total – phase I and II** n	**Positivity rate** i	N/A	76.9% (30/39)	11.1% (1/9)	N/A	65.0% (39/60)		N/A	65.7% (23/35)	8.3% (1/12)	N/A	68.7% (92/134)	19.0% (8/42)	74.6% (100/134)
	**Case confirmation rate** j	N/A	28.1% (23/82)	0% (0/82)	N/A	40.2% (33/82)	2.4% (2/82)	N/A	7.3% (6/82)	0% (0/82)	N/A	75.6% (62/82)	2.4% (2/82)	78.1% (64/82)

Table 5 shows results of external quality assurance for PCR. During the initial phase (phase I) PCR samples were analyzed at DITM by IS*2404* standard PCR and IS*2404* quantitative real-time PCR (qPCR). During the transitional phase (phase II) diagnostic sample pairs where analyzed in parallel at INH (IS*2404* dry-reagent-based [DRB] PCR) and DITM as described for phase I. Positivity rates and case confirmation rates are provided for IS*2404* DRB- and standard PCR, and additional diagnostic yields were calculated for IS*2404* qPCR. N/A, not applicable.

a Swab samples, DNA extract were prepared from swab samples.

b FNA samples, DNA extract were prepared from fine-needle aspirate samples.

c Punch samples, DNA extract were prepared from 3-mm punch biopsy samples.

d Total per phase and laboratory.

e INH applied IS*2404*-DRB-PCR. [21]

f DITM applied standard, gel-based, IS*2404* PCR [17] and IS*2404* qPCR [27], [42] on all DNA extracts tested negative with standard PCR. For qPCR the additional diagnostic yield (i.e. the deviation of total final result from total result of standard PCR) were 5.7% (phase I) and 6.1% (phase II).

g Final result of standard PCR and qPCR.

h Phase I, initial phase of implementation of the national reference laboratory at INH from September through December 2010.

i Positivity rate, number of positive samples divided by the total number of samples tested.

j Case confirmation rate, number of laboratory confirmed BUD patients divided by the total number of suspected BUD cases.

k Rate of false negative results at INH as determined by re-testing of DNA extracts at DITM by standard PCR.

l Rate of concordant results from sample pairs at INH and DITM.

m Phase II, transitional phase of implementation of the national reference laboratory at INH from January 2011 through April 2012.

n Total results of the initial and the transitional phase.

### Laboratory confirmed patients

The case confirmation rates for microscopy were 31.3% (5/16) at CHR and 37.5% (6/16) at DITM during the initial phase, and 43.9% (29/66) at CHR, 47.0% (31/66) at INH, and 53.0% (35/66) at DITM during the transitional phase. In total 50.0% (41/82) of the suspected BUD cases were confirmed by microscopy. (Table 3)

The case confirmation rates for PCR were 75.0% (12/16) at DITM during the initial phase, and 71.2% (47/66) at INH and 78.8% (52/66) at DITM (including two cases additionally confirmed by qPCR) during the transitional phase. In total 78.1% (64/82) of the suspected BUD cases were confirmed by PCR. (Table 5)

### Epidemiological baseline and treatment data of confirmed BUD cases

**Figure 2:**
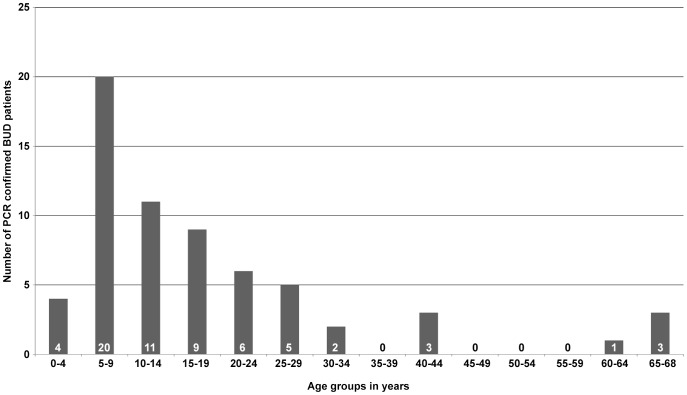
Age distribution of laboratory confirmed BUD patients. Age distribution of 64 laboratory confirmed BUD patients recruited from September 2010 through April 2012. The age of all patients was known and 48.4% (31/64) were in age group 5–14 years. The age range was 2–68 years with a mean of 18.1 years and a median of 13 years.

Out of 64 laboratory confirmed BUD patients, 51.6% (33/64) had non-ulcerative lesions (plaque, n = 17; nodule, n = 10; papule, n = 1; edema, n = 5) and 48.4% (31/64) had ulcerative lesions, 48.4% (31/64) were male, and 48.4% (31/64) were in age group 5–14 years (age range 2–68 years, mean 18.1 years, median 13 years). Figure 2

The confirmed BUD patients originated from four districts of region “Maritime” (Yoto, n = 37; Zio, n = 22; Vo, n = 1; Golfe, n = 1), two districts of region “Plateaux” (Anié, n = 1; Ogou, n = 1) and one district of region “Savanes” (Dapaong, n = 1). The categories of lesions according to WHO classification [3] were as follows: 43.8% (28/64) category I, 40.6% (26/64) category II and 15.6% (10/64) category III. (Table 1)

All patients with suspected BUD (n = 82) who presented in Togo during the study period were included (no refusals to participate) and clinical samples were collected and analyzed from all of them. All laboratory confirmed BUD patients (n = 64) received a full course of treatment with rifampicin and streptomycin; in addition, six patients, despite negative laboratory results, were subjected to antimycobacterial treatment based on strong clinical suspicion of BUD. Although no regular outreach activities were conducted in region ”Plateaux” and ”Savanes“ patients from both regions were referred to CHR for treatment.

### Impact on quality of BUD control

The number of patients with non-ulcerative lesions among all PCR-confirmed patients increased significantly (*p*<0.01) from 37.0% (as determined for the study cohort from 2007–2010, 119 patients) to 50.0% (current study cohort from January 2011 through April 2012, 52 patients).

Compared to the previous study category I lesions increased from 36.9% (95% CI: 28.3–45.6) to 44.2% (95% CI: 30.7–57.7), category II lesions increased from 32.8% (95% CI: 24.3–41.2) to 36.6% (95% CI: 23.5–49.6) and category III lesions decreased from 30.3% (95% CI: 22.0–38.5) to 19.2% (95% CI: 8.5–29.9).

The mean duration of disease before clinical diagnosis decreased from 51.8 (95% CI: 19.0–84.7) to 35.0 (95% CI: 23.5–46.5) days (no significant difference) among patients with non-ulcerative lesions, and significantly from 182.6 [95% CI: 119.2–245.9] to 82.1 [95% CI: 51.3–112.8] days among patients with ulcerative lesions. (Table 6)

**Table 6:** Impact of local reference laboratory and external quality assurance measures on BUD control.

Year of clinical presentation	2007	2008	2009	2010	2011	2012	2007–2010	2011–2012
**No. of confirmed BUD patients** a	10	38	33	38	41	11	119	52
No. of confirmed patients with non-ulcerative lesion	3	6	12	23	21	5	44	26
No. of confirmed patients with ulcerative lesions	7	32	21	15	20	6	75	26
Rate of confirmed BUD patients with non-ulcerative lesions^b^	30.0% (3/10)	15.8% (6/38)	36.4% (12/33)	60.5% (23/38)	51.2% (21/41)	45.5% (5/11)	37.0% (44/119)	50.0% (26/52)
**Rate of confirmed BUD patients – category I** c	20.0% (2/10)	50.0% (19/38)	24.2% (8/33)	39.5% (15/38)	46.3% (19/41)	36.4% (4/11)	36.9% (44/119)	44.2% (23/52)
95% confidence interval	0–44.8	34.1–65.9	9.6–38.9	23.9–55.0	31.1–61.6	7.9–64.8	28.3–45.6	30.7–57.7
**Rate of confirmed BUD patients – category II** d	30.0% (3/10)	36.8% (14/38)	30.3% (10/33)	31.6% (12/38)	34.2% (14/41)	45.5% (5/11)	32.8% (39/119)	36.6% (19/52)
95% confidence interva	1.6–58.4	21.5–52.2	14.6–46.0	16.8–46.4	19.6–48.7	16.0–74.9	24.3–41.2	23.5–49.6
**Rate of confirmed BUD patients – category III** e	50.0% (5/10)	13.2% (5/38)	45.5% (15/33)	29.9% (11/38)	19.5% (8/41)	18.1% (2/11)	30.3% (36/119)	19.2% (10/52)
95% confidence interval	19.0–81.0	2.4–23.9	28.5–62.4	14.5–43.4	7.4–31.6	0–41.0	22.0–38.5	8.5–29.9
**Mean duration of disease before clinical diagnosis in days** f								
Patients with non-ulcerative lesions	318.7	74.0	25.8	24.8	30.3	54.6	51.8	35.0
95% confidence interval	0–718.2	16.7–131.4	12.6–38.9	16.6–33.1	18.7–41.9	23.2–86.0	19.0–84.7	23.5–46.5
Patients with ulcerative lesions	386.0	239.2	107.6	71.8	87.5	64.0	182.6	82.1
95% confidence interval	78.3–693.7	118.2–360.1	59.6–55.6	45.6–98.0	48.0–27.0	45.0–83.0	119.2–245.9	51.3–112.8
All patients	365.8	213.1	77.8	43.4	58.2	59.7	134.2	58.5
95% confidence interval	130.8–600.8	109.3–316.9	44.3–111.3	29.9–56.8	36.4–80.0	42.8–76.6	91.1–177.4	41.1–76.0

Table 6 shows analyses of clinical parameters (i.e. “type of lesion” and “duration of disease before clinical diagnosis”) among PCR confirmed BUD new cases to assess impact of the local reference laboratory and external quality assurance measures on BUD control in Togo. Therefore, data from a previous study (September 2007 through December 2010) prior to implementation of the national reference laboratory at INH were analyzed and compared with data obtained in the present study (January 2011 through April 2012). Analysis for linear trends in proportions revealed a significant (*p*<0.01) increase of patients presenting with non-ulcerative lesions from 37.0% (2007–2010) to 50.0% (2011–2012). The mean duration of disease among patients with non-ulcerative lesions before presentation and establishment of clinical diagnosis decreased not significantly from 51.8 (95% CI: 19.0–84.7) to 35.0 (95% CI: 23.5–46.5) days during the two observation periods. However, the mean duration of disease among patients with ulcerative lesions before presentation of patients and establishment of clinical diagnosis decreased significantly from 182.6 (95% CI: 119.2–245.9) to 82.1 (95% CI: 51.3–112.8) days during the two observation periods. Furthermore, analysis of the development of categories of lesions showed a statistically non significant decrease from 30.3% (95% CI: 22.0–38.5) to 19.2% (95% CI: 8.5–29.9) of category III lesions. BUD, Buruli ulcer disease; CI, confidence interval.

a Number of confirmed BUD patients, laboratory confirmation was conducted by standard IS*2404* PCR, IS*2404* DRB-PCR and/or IS*2404* qPCR.

b Rate of confirmed BUD patients with non-ulcerative lesions among all confirmed BUD patients per observation period.

c Category I, single lesion <50 mm in diameter.

d Category II, single lesion between 50 and 150 mm in diameter.

e Category III, single lesion >150 mm in diameter or multiple lesions, osteomyelitis or lesions at critical sites.

f Mean duration of disease in days based on the time from first recognition of clinical symptoms by patients and availability of the clinical diagnosis “BUD”. Only data from PCR confirmed BUD patients were analyzed.

## Discussion

Laboratory confirmation of suspected BUD cases, in particular by molecular diagnostic tests, plays a crucial role for clinical management, disease control and research on *M. ulcerans*.

To achieve the targeted PCR confirmation rate of more than 50% of suspected BUD cases worldwide, WHO has set up a network of external and local PCR reference laboratories [36]. Whereas until the early 2000s laboratory diagnostic services for endemic countries were mainly provided by external reference laboratories, until 2011 six African countries (Ivory Coast, Ghana, Benin, Cameroon, Central African Republic, Democratic Republic of Congo) installed their own reference laboratories upon increasing demand for local diagnostic capacity [6], [10], [11], [18], [20]–[26], [29], [30], [32], [37], [44]–[46]. Due to the absence of laboratory facilities a number of countries still require support from external reference laboratories; in general however, the role of external reference laboratories has shifted to development of improved laboratory techniques for application in endemic countries, technical support and training of local laboratory staff, as well as external quality assurance for newly established reference laboratories [6], [11], [21], [23]–[32], [37]–[40], [43].

As well known from other studies, the implementation of reference level laboratory facilities necessitates multiple provisions in terms of logistics, trained personnel and quality management [11], [23], [47], [48]. In the case of Togo, extensive preparatory work conducted in the context of previous research projects by DAHWT and DITM [13], vast expertise gained from a longstanding cooperation with partners in Ghana [21], [23], [25], [26], [29], [40], as well as continuous exchange of information with other “BuruliVac” partners [6], [32] facilitated the implementation of a national reference laboratory considerably.

Excellent technical skills of INH laboratory staff in conventional and molecular microbiological diagnostic techniques allowed starting laboratory training at an advanced level. All training activities took place at INH; basic laboratory training according to the concept of short-term “training of trainers” workshops in Europe as successfully applied by other external reference laboratories was not required.

In consideration of the existing quality management systems at DITM and INH, special emphasis was given to standardization of all relevant procedures. SOPs defined the interaction of the laboratory with external partners in the field and the external reference laboratory in Germany, as well as all processes within the laboratory, and granted a smooth workflow from the beginning of the project. Standardized documentation of all analyses and results in standardized laboratory forms and the project-specific web-based database facilitated rapid retracing of errors for local and external reference laboratory and allowed targeted training measures.

To measure the quality of diagnostics conducted at INH, we determined concordance rates between local and external reference laboratories. Compared to a previous study [13], the concordance rate for microscopic analysis between CHR and DITM (initial and transitional phase) increased from less than 70% to 90%, and the concordance rate between INH and DITM was over 90% during the transitional phase, suggesting a high standard of microscopy at both, CHR and INH. Compared to previous findings [13], also the case confirmation rate for microscopy increased from 30% (CHR) to 43% (CHR) and 47% (INH), respectively. Likewise, concordance rates between INH and DITM for PCR of swab and punch biopsy samples were over 95%. In this study, instead of testing the same sample subsequently at both laboratories, sample pairs were collected and one sample each was sent to DITM and INH to allow quality control for both, extraction efficiency and amplification. As already observed in other studies, parallel samples – even if collected from the same site of the lesion - may show an inhomogeneous distribution of mycobacteria and may increase the normal inter-laboratory variation regularly observed for weakly positive samples ([23], [49], unpublished data). Therefore, the findings suggest high quality of PCR conducted at INH. With 93% the inter-laboratory concordance rate for FNA samples was slightly lower which may be attributable to dividing FNA samples in two pieces for microscopy and PCR at INH (whereas the entire parallel sample was subjected to PCR at DITM). Consequently, also the case confirmation rate at INH was a little lower (71%) than at DITM (76%). Future EQA of PCR diagnostics is conducted on DNA extracts only, therefore both confounders (sample pairs and divided samples) are excluded.

In addition to conventional gel-based PCR, DITM applied IS*2404* qPCR on negative samples which resulted in laboratory confirmation of two additional cases. As real-time PCR facilities are available at INH, implementation of IS*2404* qPCR is envisaged for 2013. Laboratories in endemic countries without access to real-time PCR may consider forwarding at least samples from patients with strong clinical suspicion but negative conventional PCR result to an external reference laboratory for confirmatory IS*2404* qPCR.

The study also attempted to measure the impact of local laboratory capacity and quality management on BUD control. The increase of the rate of non-ulcerative lesions by 13%, the significant reduction of the diagnostic delay by more than 100 days for patients with ulcerative lesions as compared to a previous study [13] and the reduction of category III lesions from 30.3% to 19.2% may be attributed to an extended quality management system also comprising patient related procedures outside the laboratory and intensified training measures.

Already during the previous study period from 2007 through 2010 CLTs, ICPs, ASCs and other field staff had been trained in 28 workshops with 152 participants. Since 2011, however, training measures achieved a roughly five-fold increase in coverage, and training of teams instead of individuals resulted in a multiplier effect in terms of knowledge transfer which became noticeable also in areas without regular outreach activities through referral of patients to CHR. The availability of trained CLT teams in 11 districts, in particular the ASCs, increased the coverage of sensitization activities and allowed to conduct extensive “information, education and communication” (IEC) campaigns under the guidance of DAHWT and PNLUB-LP in regions “Maritime” and “Central” accompanied by regular outreach activities to identify suspected BUD cases in the field. Finally, supervision of CLT teams by the CHR BUD team in terms of re-examining these patients provided continuous on-site training for CLT teams and enhanced the diagnostic skills of all field staff involved. Feed- back of laboratory results through a newly established reporting chain from INH to community level not only provides the basis for targeted case finding activities in the environment of confirmed patients, but is also conceived as confidence-building measure by ASCs as well as patients and their families. Altogether, the outreach system implemented in 2011 allowed to realize key components of BUD control in the field of early case detection, diagnosis and treatment as defined by the WHO [7], and more than 90% of BUD cases are currently detected through active case finding (opposed to roughly 60% in the previous study).

Whereas these outreach activities resulted in a constant flow of diagnostic samples from suspected BUD cases from peripheral health facilities in region “Maritime” via the regional hospital (CHR) to INH, and the first cases from region “Plateaux” and “Savanes” have been identified, to date no cases from region “Central” have been confirmed.

Since June 2012, a cooperation agreement between the “Faculté Mixte de Médécine et de Pharmacie” of the University of Lomé, Togo and the Faculty of Medicine of the Ludwig-Maximilians-University, Munich, Germany, has reinforced the existing diagnostic network through initiation of a collaboration with the “Laboratoire de Biologie Moléculaire et d'Immunologie” (BIOLIM), “Département des Sciences Fondamentales et Biologiques”. BIOLIM will support ongoing EQA measures in the field of quality control, academic and in-service training of local laboratory staff, thus contribute to maintaining sustainable standards in laboratory confirmation of BUD. Furthermore, access to a nationwide laboratory network established in the context of research on HIV and other infectious diseases conducted by BIOLIM will enable operational research on decentralised diagnostics and increase the efficiency of BUD control. [7], [48], [50]

## Supporting Information

Form S1 BuruliVac laboratory data entry form.
(PDF)

Forms S2 BuruliVac MIC result forms – CHR, Tsévié, and INH, Lomé.
(PDF)

SOP S1 Collection, transport and storage of diagnostic specimens.
(PDF)

SOP S2 Microscopic analysis for the detection of acid fast bacilli.
(PDF)

SOP S3 Extraction of mycobacterial DNA from clinical specimens.
(PDF)

SOP S4 IS*2404*-DRB-PCR for detection of *M. ulcerans* DNA.
(PDF)

## References

[1] Stienstra Y, van Roest MH, van Wezel MJ, Wiersma IC, Hospers IC, et al (2005) Factors associated with functional limitations and subsequent employment or schooling in Buruli ulcer patients. Trop Med Int Health 10 12: 1251–7. 16359405 10.1111/j.1365-3156.2005.01519.x

[2] World Health Organization (2006) Buruli Ulcer: Prevention of Disability (POD). Geneva: World Health Organization.

[3] World Health Organization (2008) Buruli ulcer: progress report, 2004–2008. Wkly Epidemiol Rec 83: 145–56. 18437758

[4] Barogui Y, Johnson RC, van der Werf TS, Sopoh G, Dossou A, et al (2009) Functional limitations after surgical or antibiotic treatment for Buruli ulcer in Benin. Am J Trop Med Hyg 81 1: 82–7. 19556571

[5] Schunk M, Thompson W, Klutse E, Nitschke J, Opare-Asamoah K, et al (2009) Outcome of patients with buruli ulcer after surgical treatment with or without antimycobacterial treatment in Ghana. Am J Trop Med Hyg 81 1: 75–81. 19556570

[6] Phanzu DM, Suykerbuyk P, Imposo DB, Lukanu PN, Minuku JB, et al (2011) Effect of a control project on clinical profiles and outcomes in buruli ulcer: a before/after study in Bas-Congo, Democratic Republic of Congo. PLoS Negl Trop Dis 5 12: e1402. 22216362 10.1371/journal.pntd.0001402 PMC3246436

[7] World Health Organization (2007) Summary report of the WHO annual meeting on Buruli ulcer, 2–4 April 2007 and Report of the Technical Advisory Group (TAG) meeting, 5 April 2007. Geneva: World Health Organization.

[8] Johnson PD, Hayman JA, Quek TY, Fyfe JA, Jenkin GA, et al (2007) Consensus recommendations for the diagnosis, treatment and control of Mycobacterium ulcerans infection (Bairnsdale or Buruli ulcer) in Victoria, Australia. Med J Aust 186 2: 64–8. 17223765 10.5694/j.1326-5377.2007.tb00802.x

[9] World Health Organization (2008) Meeting of the WHO Technical Advisory Group on Buruli ulcer, 3 April 2008, Geneva, Summary Report. Geneva: World Health Organization.

[10] Mensah-Quainoo E, Yeboah-Manu D, Asebi C, Patafuor F, Ofori-Adjei D, et al (2008) Diagnosis of Mycobacterium ulcerans infection (Buruli ulcer) at a treatment centre in Ghana: a retrospective analysis of laboratory results of clinically diagnosed cases. Trop Med Int Health 13: 191–8. 18304265 10.1111/j.1365-3156.2007.01990.x

[11] Affolabi D, Tanimomo-Kledjo B, Anyo G, Johnson RC, Anagonou SY, et al (2008) Setting up a national reference laboratory for Buruli ulcer: the case of Benin. Trop Med Int Health 13 3: 365–8. 18397399 10.1111/j.1365-3156.2008.02011.x

[12] Beissner M, Herbinger KH, Bretzel G (2010) Laboratory diagnosis of Buruli ulcer disease. Future Microbiol 5 3: 363–70. 20210548 10.2217/fmb.10.3

[13] Bretzel G, Huber KL, Kobara B, Beissner M, Piten E, et al (2011) Laboratory confirmation of Buruli ulcer disease in Togo, 2007–2010. PLoS Negl Trop Dis 7: e1228. 10.1371/journal.pntd.0001228 PMC3139659 21811641

[14] Portaels F, Agular J, Fissette K, Fonteyne PA, De Beenhouwer H, et al (1997) Direct detection and identification of Mycobacterium ulcerans in clinical specimens by PCR and oligonucleotide-specific capture plate hybridization. J Clin Microbiol 35: 1097–100. 9114387 10.1128/jcm.35.5.1097-1100.1997 PMC232709

[15] Ross BC, Marino L, Oppendisano F, Edwards R, Robins-Browne RM, et al (1997) Development of a PCR Assay for rapid Diagnosis of Mycobacterium ulcerans Infection. J Microbiol Clin 35 7: 1696–700. 10.1128/jcm.35.7.1696-1700.1997 PMC229824 9196176

[16] Guimaraes-Peres A, Portaels F, de Rijk P, Fissette K, Pattyn SR, et al (1999) Comparison of two PCRs for detection of Mycobacterium ulcerans. J Clin Microbiol 37: 206–8. 9854092 10.1128/jcm.37.1.206-208.1999 PMC84208

[17] Stinear T, Ross BC, Davies JK, Marino L, Robins-Browne RM, et al (1999) Identification and characterization of IS2404 and IS2606: two distinct repeated sequences for detection of Mycobacterium ulcerans by PCR. J Clin Microbiol 37 4: 1018–23. 10074520 10.1128/jcm.37.4.1018-1023.1999 PMC88643

[18] Stienstra Y, van der Werf TS, Guarner J, Raghunathan PL, Spotts Whitney EA, et al (2003) Analysis of an IS2404-based nested PCR for diagnosis of Buruli ulcer disease in regions of Ghana where the disease is endemic. J Clin Microbiol 41: 794–7. 12574285 10.1128/JCM.41.2.794-797.2003 PMC149660

[19] Rondini S, Mensah-Quainoo E, Troll H, Bodmer T, Pluschke G, et al (2003) Development and application of real-time PCR assay for quantification of Mycobacterium ulcerans DNA. J Clin Microbiol 41 9: 4231–7. 12958250 10.1128/JCM.41.9.4231-4237.2003 PMC193839

[20] Noeske J, Kuaban C, Rondini S, Sorlin P, Ciaffi L, et al (2004) Buruli ulcer disease in Cameroon rediscovered. Am J Trop Med Hyg 70 5: 520–6. 15155984

[21] Siegmund V, Adjei O, Racz P, Berberich C, Klutse E, et al (2005) Dry-reagent-based PCR as a novel tool for laboratory confirmation of clinically diagnosed Mycobacterium ulcerans-associated disease in areas in the tropics where M. ulcerans is endemic. J Clin Microbiol 43 1: 271–6. 15634982 10.1128/JCM.43.1.271-276.2005 PMC540149

[22] Phillips R, Horsfield C, Kuijper S, Lartey A, Tetteh I, et al (2005) Sensitivity of PCR targeting the IS2404 insertion sequence of Mycobacterium ulcerans in an Assay using punch biopsy specimens for diagnosis of Buruli ulcer. J Clin Microbiol 43 8: 3650–6. 16081892 10.1128/JCM.43.8.3650-3656.2005 PMC1233911

[23] Bretzel G, Siegmund V, Nitschke J, Herbinger KH, Thompson R, et al (2006) External quality assurance for the laboratory diagnosis of Buruli ulcer disease in Ghana. Trop Med Int Health 11 11: 1688–93. 17054748 10.1111/j.1365-3156.2006.01722.x

[24] Phanzu DM, Bafende EA, Dunda BK, Imposo DB, Kibadi AK, et al (2006) Mycobacterium ulcerans disease (Buruli ulcer) in a rural hospital in Bas-Congo, Democratic Republic of Congo, 2002–2004. Am J Trop Med Hyg 75 2: 311–4. 16896139

[25] Siegmund V, Adjei O, Nitschke J, Thompson W, Klutse E, et al (2007) Dry reagent-based polymerase chain reaction compared with other laboratory methods available for the diagnosis of Buruli ulcer disease. Clin Infect Dis 45 1: 68–75. 17554703 10.1086/518604

[26] Bretzel G, Siegmund V, Nitschke J, Herbinger KH, Thompson W, et al (2007) A stepwise approach to the laboratory diagnosis of Buruli ulcer disease. Trop Med Int Health 12 1: 89–96. 17207152 10.1111/j.1365-3156.2006.01761.x

[27] Fyfe JA, Lavender CJ, Johnson PD, Globan M, Sievers A, et al (2007) Development and application of two multiplex real-time PCR assays for the detection of Mycobacterium ulcerans in clinical and environmental samples. Appl Environ Microbiol 73 15: 4733–40. 17526786 10.1128/AEM.02971-06 PMC1951036

[28] Affolabi D, Bankolé H, Ablordey A, Hounnouga J, Koutchakpo P, et al (2008) Effects of grinding surgical tissue specimens and smear staining methods on Buruli ulcer microscopic diagnosis. Trop Med Int Health 13 2: 187–90. 18304264 10.1111/j.1365-3156.2007.01989.x

[29] Herbinger KH, Adjei O, Awua-Boateng NY, Nienhuis WA, Kunaa L, et al (2009) Comparative study of the sensitivity of different diagnostic methods for the laboratory diagnosis of Buruli ulcer disease. Clin Infect Dis 48: 1055–64. 19275499 10.1086/597398

[30] Kibadi K, Boelaert M, Fraga AG, Kayinua M, Longatto-Filho A, et al (2010) Response to treatment in a prospective cohort of patients with large ulcerated lesions suspected to be Buruli Ulcer (Mycobacterium ulcerans disease). PLoS Negl Trop Dis 4 7: e736. 20625556 10.1371/journal.pntd.0000736 PMC2897843

[31] Yeboah-Manu D, Asante-Poku A, Asan-Ampah K, Ampadu ED, Pluschke G, et al (2011) Combining PCR with microscopy to reduce costs of laboratory diagnosis of Buruli ulcer. Am J Trop Med Hyg 2011 Nov;85 5: 900–4. 10.4269/ajtmh.2011.11-0362 PMC3205638 22049046

[32] Affolabi D, Sanoussi N, Vandelannoote K, Odoun M, Faïhun F, et al (2012) Effects of decontamination, DNA extraction, and amplification procedures on the molecular diagnosis of Mycobacterium ulcerans disease (Buruli ulcer). J Clin Microbiol 50 4: 1195–8. 22259213 10.1128/JCM.05592-11 PMC3318535

[33] Meyers WM, Tignokpa N, Priuli GB, Portaels F (1996) Mycobacterium ulcerans infection (Buruli ulcer): first reported patients in Togo. Br J Dermatol 134: 1116–21. 8763437

[34] Songné B, Abete B, Scotte M, Tignokpa N, Valenti P (2001) [Buruli ulcer in Togo: 21 cases]. Presse Med 30: 533. 11317927

[35] James K, Attipou KK, James YE, Blakime M, Tignokpa N, et al (2003) [Buruli ulcer in Togo: a hospital study]. Sante 13: 43–47. 12925323

[36] World Health Organization, Laboratory Support Network. Global network of laboratories for confirming Mycobacterium ulcerans infection (Buruli ulcer). Geneva: World Health Organization. Available: http://www.who.int/buruli/Global_network_laboratories_PCR.pdf. Accessed 16 July 2012.

[37] Herbinger KH, Brieske D, Nitschke J, Siegmund V, Thompson W, et al (2009) Excision of pre-ulcerative forms of Buruli Ulcer Disease: A curative treatment? Infection 14 37: 20–5. 10.1007/s15010-008-8073-4 19139811

[38] Eddyani M, Fraga AG, Schmitt F, Uwizeye C, Fissette K, et al (2009) Fine needle aspiration, an efficient sampling technique for the bacteriological diagnosis of nonulcerative Buruli ulcer lesions. J. Clin. Microbiol 47: 1700–04. 10.1128/JCM.00197-09 PMC2691074 19386847

[39] Phillips RO, Sarfo FS, Osei-Sarpong F, Boateng A, Tetteh I, et al (2009) Sensitivity of PCR targeting Mycobacterium ulcerans by use of fine-needle aspirates for diagnosis of Buruli ulcer. J Clin Microbiol 47: 924–26. 19204098 10.1128/JCM.01842-08 PMC2668351

[40] Herbinger KH, Beissner M, Huber K, Awua-Boateng NY, Nitschke J, et al (2010) Efficiency of fine-needle aspiration compared with other sampling techniques for laboratory diagnosis of Buruli ulcer disease. J Clin Microbiol 48 10: 3732–4. 20739480 10.1128/JCM.01549-10 PMC2953090

[41] World Health Organization (2010) Guidance on sampling techniques for laboratory-confirmation of Mycobacterium ulcerans infection (Buruli ulcer disease). Geneva: World Health Organization.

[42] World Health Organization (2001) Diagnosis of Mycobacterium ulcerans disease. Geneva: World Health Organization.

[43] Beissner M, Symank D, Phillips RO, Amoako YA, Awua-Boateng NY, et al (2012) Detection of Viable Mycobacterium ulcerans in Clinical Samples by a Novel Combined 16S rRNA Reverse Transcriptase/IS2404 Real-Time qPCR Assay. PLoS Negl Trop Dis 6 8: e1756. 22953006 10.1371/journal.pntd.0001756 PMC3429398

[44] Debacker M, Aguiar J, Steunou C, Zinsou C, Meyers WM, et al (2004) Mycobacterium ulcerans disease (Buruli ulcer) in rural hospital, Southern Benin, 1997–2001. Emerg Infect Dis 10 8: 1391–8. 15496239 10.3201/eid1008.030886 PMC3320395

[45] Coulibaly B, Coulibaly-N'Golo MD, Ekaza E, Aka N, N'Guessan KR, Baudryard A, et al (2010) [Implementation of in vitro culture of Mycobacterium ulcerans from clinical samples versus detection of acid-fast bachilli and bacterial genome in Abidjan, Côte d'Ivoire]. Bull Soc Pathol Exot 103 1: 2–7. 20084485 10.1007/s13149-009-0002-y

[46] Minime-Lingoupou F, Beyam N, Zandanga G, Manirakiza A, N'Domackrah A, et al (2010) Buruli ulcer, Central African Republic. Emerg Infect Dis 16 4: 746–8. 20350416 10.3201/eid1604.090195 PMC3321933

[47] APHL, CDC, IUATLD, KNCV, RIT & WHO (2002) External quality assessment of AFB smear microscopy. APHL, Washington D.C.

[48] WHO (2004) Resolution WHA57.1. Surveillance and control of Mycobacterium ulcerans disease (Buruli ulcer). In: Fifty-seventh World Health Assembly, Geneva, 17–22 May 2004. Resolutions and decisions Geneva: World Health Organization. WHA57/2004/REC/1:1–2.

[49] Kiefer T, Hirt C, Schüler F, Busemann C, Wodny M, et al (2012) Statistical analysis of results obtained by real-time PCR for improvement of absolute quantification of target sequences. Clin Lab 58 5–6: 465–70. 22783576

[50] WHO (2009) Cotonou Declaration on Buruli Ulcer. Geneva: World Health Organization.

