# Supplementary material for: Implementation of a National Reference Laboratory for Buruli Ulcer Disease in Togo
Source: PLoS Negl Trop Dis. 2013 Jan 24;7(1):e2011. doi: 10.1371/journal.pntd.0002011 (PMC3554568; doi:10.1371/journal.pntd.0002011)
Supplement: Form S1 — BuruliVac laboratory data entry form. (PDF) [file pntd.0002011.s001.pdf]

BURULIVAC **Country: Togo** **Suspected BUD case**

Laboratory Data Entry Form

Date ....201.....................

Use separate form for each "visit" and each lesion (if multiple lesions) per patient!

A: Patient ID .... **Hospital** ....

Family name .... **First name** ....

Village .... **District** ....

Age ....years **Sex** ☐ m ☐ f **BCG Scar** ☐ yes ☐ no

B: Classification ☐ **New case** ☐ **Recurrence**

C: Clinical Presentation **&** **Number of lesions**

☐ Nodule ☐ Papule ☐ Plaque ☐ Single lesion

☐ Ulcer ☐ Edema ☐ Osteomyelitis or ☐ Multiple lesions (...............)

D: Visit (Time of sample collection)

☐ pre-treatment (V1) ☐ other: week..............

E: Duration of Disease .... day(s) .... week(s) .... month(s)

F: Location of the Lesion

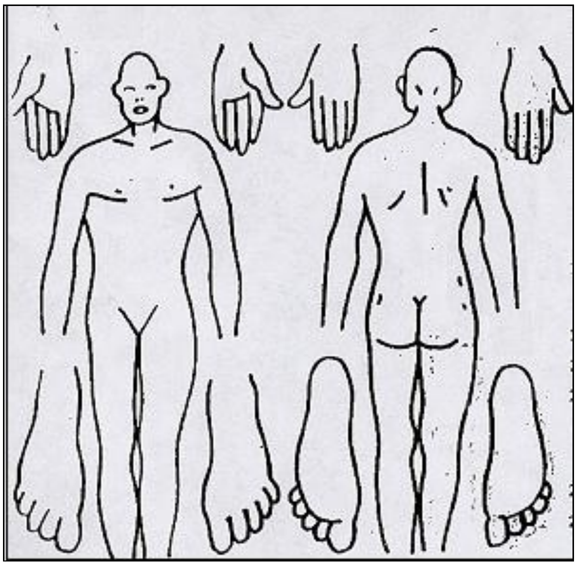

Dosage of Rifampicin :......................mg/d

Dosage of Streptomycin : ....g/d

Dosage of other (name) : ....

Treatment Start Date : ....

(Photographical) documentation:

☐ no ☐ yes, by:..

BurulíVac **BURULIVAC** **Country: Togo** **Suspected BUD case**

G: Clinical samples

Nodule, Papule, Plaque, Edema

Ø of lesion ..../.................... **mm** **Category**....................................

MIC (CHR/INH/DITM) + PCR (INH)* - PCR (INH)** - PCR (DITM)***		
**FNA*** O	**Punch biopsy**** O	**Tissue surgery**** O
**FNA***** O	**Punch biopsy****** O	**Tissue surgery****** O

Ulcer

Ø of lesion ..../.......... .... **mm** **Category**....................................

MIC (CHR/INH/DITM)* - MIC (CHR/INH/DITM) + PCR (INH)** - PCR (INH)*** PCR (DITM)****		
**Swab*** O	**FNA**** O	**Punch biopsy****** O
**Swab****** O	**FNA******* O	**Punch biopsy******* O
**Swab******* O		

H: Remarks
