# Supplementary material for: Implementation of a National Reference Laboratory for Buruli Ulcer Disease in Togo
Source: PLoS Negl Trop Dis. 2013 Jan 24;7(1):e2011. doi: 10.1371/journal.pntd.0002011 (PMC3554568; doi:10.1371/journal.pntd.0002011)
Supplement: Forms S2 — BuruliVac MIC result forms – CHR, Tsévié, and INH, Lomé. (PDF) [file pntd.0002011.s002.pdf]

MIC résultat	BuruliVac – Togo<br>CHR Tsévié

No ID BuruliVac d'échantillon : ______________________________

Nom du patient :

Date d'arrivée :

Date de la lecture :

**Résultat**	**Code**
Plus de 10 BAAR / champ au moins dans 20 champs	+++ (positive)
1 – 10 BAAR / champ	++ (positive)
10 – 99 BAAR / 100 champs	+ (positive)
1-9 BAAR / 100 champs	Marquer le nombre exact/ 100 champs (positive)
Aucun BAAR dans au moins 100 champs	- (negative)

CHR – Tsévié

1ier lecture

☐ **3+** ☐ **2+** ☐ **1+** ☐ **Nombre exact :** ☐ **neg**

2ième lecture (optionnel)

☐ 3+ ☐ 2+ ☐ 1+ ☐ Nombre exact : ☐ neg

3ième lecture (si les résultats de la 1ère et 2ième lecture sont discordants)

☐ 3+ ☐ 2+ ☐ 1+ ☐ Nombre exact : ☐ neg

Résultat final

☐ **3+** ☐ **2+** ☐ **1+** ☐ **Nombre exact :** ☐ **neg**

☐ **Lecture impossible**

Raison:

☐ **Nouvelle lame demandé**

Commentaire :

# MIC résultat	BuruliVac – Togo<br>INH Lomé

No ID BuruliVac d'échantillon : ______________________________

Nom du patient :

Date d'arrivée :

Date de la lecture :

**Résultat**	**Code**
Plus de 10 BAAR / champ au moins dans 20 champs	+++ (positive)
1 – 10 BAAR / champ	++ (positive)
10 – 99 BAAR / 100 champs	+ (positive)
1-9 BAAR / 100 champs	Marquer le nombre exact/ 100 champs (positive)
Aucun BAAR dans au moins 100 champs	- (negative)

Institut National d'Hygiéne, Lomé: EQA, 1ère étape

1ère lecture

☐ **3+** ☐ **2+** ☐ **1+** ☐ **Nombre exact :** ☐ **neg**

2ième lecture (optionnel)

☐ 3+ ☐ 2+ ☐ 1+ ☐ Nombre exact : ☐ neg

3ième lecture (si les résultats de la 1ère et 2ième lecture sont discordants)

☐ 3+ ☐ 2+ ☐ 1+ ☐ Nombre exact : ☐ neg

Résultat final INH (EQA 1)

☐ **3+** ☐ **2+** ☐ **1+** ☐ **Nombre exact :** ☐ **neg**

Commentaire (ex: qualité de la colorisation) :

# MIC résultat Révision	BuruliVac – Togo<br>INH Lomé – CHR Tsévié

No ID BuruliVac d'échantillon : ____________________________

Nom du patient :

Date de la lecture :

Résultat	Code
Plus de 10 BAAR / champ au moins dans 20 champs	+++ (positive)
1 – 10 BAAR / champ	++ (positive)
10 – 99 BAAR / 100 champs	+ (positive)
1-9 BAAR / 100 champs	Marquer le nombre exact/ 100 champs (positive)
Aucun BAAR dans au moins 100 champs	- (negative)

Institut National d'Hygiéne, Lomé – CHR, Tsévié

Révision de l'analyse à cause des résultats discordants d'INH et du CHR, Tsévié

Résultat final d'INH et du CHR,Togo (Les deux lecteurs ensemble)

☐ **3+** ☐ **2+** ☐ **1+** ☐ **Nombre exact :** ☐ **neg**

Commentaire:
