# Supplementary material for: Implementation of a National Reference Laboratory for Buruli Ulcer Disease in Togo
Source: PLoS Negl Trop Dis. 2013 Jan 24;7(1):e2011. doi: 10.1371/journal.pntd.0002011 (PMC3554568; doi:10.1371/journal.pntd.0002011)
Supplement: SOP S1 — Collection, transport and storage of diagnostic specimens. (PDF) [file pntd.0002011.s003.pdf]

Standard Operating Procedure (SOP)

Clinical samples – suspected BUD patients

Collection, transport and storage of diagnostic specimens

1. General considerations

This document describes the standard operating procedures applied for collection, transport and storage of diagnostic specimens from suspected BUD patients enrolled in BuruliVac.

Currently WHO recommendations for diagnostic sample collection and laboratory procedures are under revision.

▲ ***Before any patient is enrolled in BuruliVac and clinical specimens are collected, ensure that informed consent has been obtained by the patient or his/her legal representative!***

2. Specimen collection

2.1 Clinical specimens for laboratory confirmation of clinically suspected BUD patients

According to the kind of lesion standardized specimen collection procedures are applied in Togo. In 2011, PCR for the laboratory diagnosis of BUD will be installed at INH Lomé and **PCR confirmation will be carried out in 3 phases:**

1) **Initial phase** – PCR is conducted at LMU Munich until PCR equipment is available at INH Lomé and local staff has been trained.
2) **Transitional phase** – PCR is conducted at DITM/LMU, Munich, and INH, Lomé, Togo, from parallel sets of specimens until PCR results of INH lab reaches >95% concordance with PCR results of DITM/LMU.
3) **Final phase** – PCR is conducted at INH, external quality assurance of PCR on DNA extracts is conducted at DITM/LMU.

According to these phases specimen collection procedures are adjusted as indicated below.

Currently, there is broad consensus among BUD experts that FNA samples are to be favoured over punch biopsies. Several aspects of patient management however, may still require the collection of punch biopsies.

Among indications that justify the collection of punch biopsies are:

- Strong clinical suspicion but negative FNA sample(s)
- Differential diagnosis
- Paradoxical reaction
- Possible recurrence (confirmation by culture)
- Treatment failure (confirmation by culture)
- Drug susceptibility testing by culture if evidence for drug resistant strains emerges
- Development of cancerous changes

<u>2.1.1 Initial phase:</u> Confirmation of clinically suspected BUD patients by PCR is conducted at DITM/LMU Munich.

During the initial phase, as long as samples are shipped to DITM/LMU, more than the minimum number of samples will be collected in order to increase the probability for positive PCR results and to avoid repeated shipping of samples.

Table 1

Nodule, Papule, Plaque, Edema

Non-surgical treatment	Surgical treatment
**specimens for MIC at CHR-TSEVIE and CHP-SOTOUBOUA (or others)**	
1st FNA	
**specimens for PCR at DITM/LMU Munich**	
2nd FNA	1 Tissue surgery
1 Punch*	

For non-ulcerative lesions preferably 2 FNA are collected per lesion.
Tissue surgery is collected at the time of surgery (if conducted for individual patients).
* Punch biopsies are only collected from patients presenting in a hospital or USP

Ulcer

Non-surgical treatment	Surgical treatment
**specimens for MIC at CHR-TSEVIE and CHP-SOTOUBOUA (or others)**	
1st Swab	1st Swab
or 1st FNA (lesion with non-undermined edge)	
**specimens for PCR at DITM/LMU Munich**	
2nd Swab	2nd Swab and 1 Tissue surgery
or 2nd FNA (lesion with non-undermined edge)	
1 Punch*	

For PCR diagnosis of ulcerative lesions with undermined edges 2 different types of samples are collected and sent to DITM/LMU: Either Swab and Punch or (in case of lesions without undermined edges) FNA and Punch
*Punch biopsies are only collected in hospitals or USP

Other (Osteomyelitis)

Non-surgical treatment	Surgical treatment
**specimens for MIC at CHR-TSEVIE and CHP-SOTOUBOUA (or others)**	
1st Swab	1st Swab
**specimens for PCR at DITM/LMU Munich**	
2nd Swab	2nd Swab and 1 Tissue surgery

Primarily 2 Swabs are collected from ulcerative lesion of osteomyelitis cases.
Tissue surgery is collected at the time of surgery.

2.1.2 Transitional phase: Confirmation of clinically suspected BUD patients by PCR is conducted at INH Lomé and DITM/LMU Munich (parallel sets of specimens tested by PCR).

Table 2

Nodule, Papule, Plaque, Edema

Non-surgical treatment	Surgical treatment
**specimens for MIC at CHR-TSEVIE and CHP-SOTOUBOUA (or others)**	
1st FNA	
**specimens for PCR at INH Lomé**	
1st FNA	1st Tissue surgery
1 Punch biopsy*	
**specimens for PCR at DITM/LMU Munich**	
2nd FNA	2nd Tissue surgery

For non-ulcerative lesions preferably FNA are collected. The 1st FNA is subjected to MIC and used for PCR at INH Lomé. The 2nd FNA is subjected to confirmatory PCR at DITM/LMU Munich.

Tissue surgery is collected at the time of surgery (if conducted for individual patients). One specimen is subjected to PCR at INH Lomé, the 2nd specimen is sent to DITM/LMU for PCR confirmation.

*Punch biopsies are only collected if – despite strong clinical suspicion – both FNAs are negative.

Ulcer

Non-surgical treatment	Surgical treatment
**specimens for MIC at CHR-TSEVIE and CHP-SOTOUBOUA (or others)**	
1st Swab	1st Swab
or 1st FNA (lesion with non-undermined edge)*	
**specimens for PCR at INH Lomé**	
2nd Swab	2nd Swab and 1st Tissue surgery
or 1st FNA (lesion with non-undermined edge)*	
1 Punch biopsy*	
**specimens for PCR at LMU Munich**	
3rd Swab	3rd Swab and 2nd Tissue surgery
or 2nd FNA (lesion with non-undermined edge)*	

For ulcerative lesions preferably Swabs are collected. The 1st Swab is subjected to MIC, the 2nd Swab is used for PCR at INH and the 3rd Swab is sent to /DITMLMU for PCR confirmation.

* FNA are collected only for lesions without undermined edges. The 1st FNA is used for MIC and PCR at INH and the 2nd FNA is sent to DITM/LMU for PCR confirmation.

Tissue surgery is collected at the time of surgery. The 1st specimen is subjected to PCR at INH and the 2nd specimen is sent to DITM/LMU for PCR confirmation.

*Punch biopsies are only collected if – despite strong clinical suspicion – both Swabs or FNAs are negative.

Other (Osteomyelitis)

Non-surgical treatment	Surgical treatment
**specimens for MIC at CHR-TSEVIE and CHP-SOTOUBOUA (or others)**	
1st Swab	1st Swab
**specimens for PCR at INH Lomé**	
2nd Swab	2nd Swab and 1st Tissue surgery
**specimens for PCR at DITM/LMU Munich**	
3rd Swab	3rd Swab and 2nd Tissue surgery

Primarily Swabs are collected from ulcerative lesion of osteomyelitis cases. The 1st Swab is subjected to MIC, the 2nd Swab is used for PCR at INH and the 3rd Swab is sent to DITM/LMU for PCR confirmation.

Tissue surgery is collected at the time of surgery. The 1st specimen is subjected to PCR at INH and the 2nd specimen is sent to DITM/LMU for PCR confirmation.

2.1.3 Final phase: Confirmation of clinical BUD subjects by PCR is conducted at INH Lomé. External quality assurance of DNA extracts is conducted at LMU, Munich.

Table 3

Nodule, Papule, Plaque, Edema

Non-surgical treatment	Surgical treatment
**specimens for MIC at CHR-TSEVIE and CHP-SOTOUBOUA (or others)**	
1st FNA	
**specimens for PCR at INH Lomé**	
2nd FNA	1 Tissue surgery
1 Punch biopsy*	

For non-ulcerative lesions preferably FNA are collected.
Tissue surgery is collected at the time of surgery (if conducted for individual patients).
*Punch biopsies are only collected if – despite strong clinical suspicion – both FNAs are negative.

Ulcer

Non-surgical treatment	Surgical treatment
**specimens for MIC at CHR-TSEVIE and CHP-SOTOUBOUA (or others)**	
1st Swab	1st Swab
or 1st FNA (lesion with non-undermined edge)*	
**specimens for PCR at INH Lomé**	
2nd Swab	2nd Swab and 1 Tissue surgery
or 2nd FNA (lesion with non-undermined edge)*	
1 Punch biopsy*	

For ulcerative lesions preferably swab samples are collected.
*FNA are collected only for lesions without undermined edges.
*Punch biopsies are only collected if – despite strong clinical suspicion – both Swabs or FNAs are negative.

Other (Osteomyelitis)

Non-surgical treatment	Surgical treatment
**specimens for MIC at CHR-TSEVIE and CHP-SOTOUBOUA (or others)**	
1st Swab	1st Swab
**specimens for PCR at INH Lomé**	
2nd Swab	2nd Swab and 1 Tissue surgery

Primarily swab samples are collected from ulcerative lesion of osteomyelitis cases.
Tissue surgery is collected at the time of surgery.

2.2.1 Specimen collection bags

Collection of diagnostic samples will apply standardized specimen collection bags. Pre-packed specimen collection bags are provided to the outreach teams and BUD clinics by DAHWT. Specimen bags contain all containers filled with transport and storage media suitable to preserve specimens for all laboratory tests (i.e. microscopy and PCR).

Each bag is labelled with the packing date of the bag to determine the expiry date of reagents. The specimen bags can be stored at room temperature for 6 months, after that period materials are replaced.

Each bag contains BuruliVac laboratory data entry forms (Form S1) and BU01 forms for data collection.

2 different types of specimen collection bags are prepared at DAHWT and provided to the BUD clinics/treatment sites and for the field for WP6:

A) Clinical specimen collection bags – non-surgical sample collection

B) Clinical specimen collection bags – surgical sample collection

▲ ***Per lesion one specimen collection bag is used!***

In the course of the three phases (see 2.1) of PCR installation at INH, specimen collection bags are adjusted accordingly. All other items needed for sampling (e.g. biopsy punches for individual cases) and the clinical management will be available in a tool box (see annex).

2.2.1.1 Content of clinical specimen collection bags for WP6 – phase I

A.I) Clinical specimen collection bags – non-surgical sample collection

1 BuruliVac data entry form
1 BU01.N form (if relapse the BU01.R form is applicable)
2 Needles for FNA, 21-gauge (G)
    alternatively 2 butterfly needles for FNA
2 Syringes (5 ml, sterile)
2 FNA containers (2 x CLS, 300 µl in 2 ml screw cap tubes)
2 Swabs
2 Swab containers (2x CLS, 700 µl in 2 ml screw cap tubes)
1 Biopsy punch (3 mm)
1 Punch container (1x CLS, 700 µl in 2 ml screw cap tube)
1 One way measuring tape
8 Etiquettes

B.I) Clinical specimen collection bags – surgical sample collection

1 BuruliVac data entry form
1 BU01.N form (if relapse the BU01.R form is applicable)
2 Swabs
2 Swab containers (2x CLS, 700 µl in 2 ml screw cap tubes)
1 Container for surgical tissue (1x CLS, 700 µl in 2 ml screw cap tube)
1 One way measuring tape
8 Etiquettes

2.2.1.2 Content of clinical specimen collection bags – phase II

A.II) Clinical specimen collection bags – non-surgical sample collection

1 BuruliVac data entry form
1 BU01.N form (if relapse the BU01.R form is applicable)
2 Needles for FNA, 21-gauge (G)
    alternatively 2 butterfly needles for FNA
2 Syringes (5 ml, sterile)
2 FNA containers (2 x CLS, 300 µl in 2 ml screw cap tubes)
3 Swabs
3 Swab containers (3x CLS, 700 µl in 2 ml screw cap tubes)
1 One way measuring tape
8 Etiquettes

B.II) Clinical specimen collection bags – surgical sample collection

1 BuruliVac data entry form
1 BU01.N form (if relapse the BU01.R form is applicable)
3 Swabs
3 Swab containers (3x CLS, 700 µl in 2 ml screw cap tubes)
2 Container for surgical tissue (2x CLS, 700 µl in 2 ml screw cap tube)
1 One way measuring tape
8 Etiquettes

2.2.1.3 Content of clinical specimen collection bags – phase III

A.III) Clinical specimen collection bags – non-surgical sample collection

1 BuruliVac data entry form
1 BU01.N form (if relapse the BU01.R form is applicable)
2 Needles for FNA, 21-gauge (G)
    alternatively 2 butterfly needles for FNA
2 Syringes (5 ml, sterile)
2 FNA containers (2x CLS, 300 µl in 2 ml screw cap tubes)
2 Swabs
2 Swab containers (2x CLS, 700 µl in 2 ml screw cap tubes)
1 One way measuring tape
8 Etiquettes

B.III) Clinical specimen collection bags – surgical sample collection

1 BuruliVac data entry form
1 BU01.N form (if relapse the BU01.R form is applicable)
2 Swabs
2 Swab containers (2x CLS, 700 µl in 2 ml screw cap tubes)
1 Container for surgical tissue (1x CLS, 700 µl in 2 ml screw cap tube)
1 One way measuring tape
8 Etiquettes

Table 4 provides detailed information on transport conditions.

Specimen	Diagnostic test	Transport medium	Transport container
Swab	DRB-PCR	700 µl CLS*	Sterile tube/cooling box
FNA	DRB-PCR	300 µl CLS	5 ml syringe/cooling box
Punch biopsy	DRB-PCR	700 µl CLS	2 ml screw cap tubes/cooling box
Tissue	DRB-PCR	700 µl CLS	2 ml screw cap tubes/cooling box

Table 4
*CLS: Cell lysis solution (Puregene, Qiagen)
For microscopy direct smears are generated on-site from the respective specimens (see table 1-3) and kept in a slide-box for transport.

2.3 Procedure of sampling

2.3.1 Diagnostic samples for laboratory diagnosis of BUD

▲***In Togo, diagnostic BUD samples will be collected by trained medical assistants under supervision of resident physicians only! Prior to specimen collection the longitudinal and transversal diameters of the lesion are determined (in mm) and registered on the laboratory data entry form.***

<u>Swabs</u>

Before a swab specimen is obtained the procedure is explained to the patients. Underneath the undermined edge the sterile swab is twirled circling the entire undermined edge of ulcers as indicated in Fig.1.

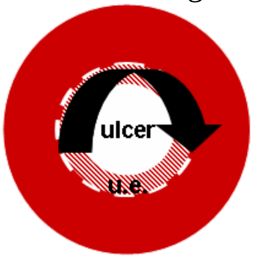

Fig. 1. Specimen collection for diagnostic swabs from ulcerative lesions with undermined edge
u.e.: undermined edge of ulcer.

The swab is placed into the respective container and labelled. Samples for PCR are placed in 2 ml screw cap tubes containing 700 µl CLS. For microscopy 1 slide is prepared on-site and stored in a slide box.

<u>FNA</u>

The procedure of FNA collection is described according to current WHO recommendations:

1. The lesion is carefully inspected and palpated.
2. The site of the lesion for doing the aspiration is determined and disinfected using an alcohol soaked swab. It is advisable to obtain the FNA from the centre of the lesion or to identify weak areas in the case of non ulcerated oedematous lesions.

3. The syringe and needle (sterile 21 G needle or preferably a butterfly needle, 2 ml or 5 ml syringe) are prepared.
4. For nodules and small plaques, the lesion is grasped with one hand and hold in a fixed and stable position.
5. Using the other hand, the needle is inserted into the estimated centre of the lesion and move back and forth (about 3 times) within the subcutaneous adipose tissue in different directions without withdrawing through the skin and suction is applied each time the needle is moved forth.
6. Finally, the needle is withdrawn. To prevent ulceration, the respective needle must not be inserted and withdrawn again.

FNA is suitable for microscopy and PCR. For PCR needle tips are inserted into the respective transport medium (CLS) and flushed to wash the aspirate into the container (note that the aspirate may not be visible) and the tube is labelled. The procedure is repeated twice. For microscopy 1 slide is prepared on-site CHR-TSEVIE and stored in a slide box.

Punch biopsy

Diagnostic tissue specimens from pre-ulcerative lesions are collected from the centre of the lesion (Fig. 2) or, in case of ulcers without undermined edges, from the necrotic margin of ulcers bordering healthy (viable) tissue (Fig. 3) using a 3 mm biopsy punch.
By determining the location of specimen collection, special attention must be paid to anatomically underlying neuro-vascular structures (especially superficial veins) in the area of the defined biopsy site. The area of specimen collection must be draped. Local anaesthesia with 2 % lidocaine is administered. The skin must be stretched perpendicular to skin tension lines, then the biopsy punch is rotated into the dermis and fully into the subcutaneous adipose tissue.
For diagnostic purposes it is mandatory that tissue specimens contain the subcutaneous adipose tissue as mycobacteria are present in that layer. The punch biopsy must be elevated above the incision level, if necessary by using sterile forceps, and excised using a sterile scalpel. The specimens are placed into the respective transport container for PCR (700µl CLS). For microscopy 1 slide is prepared at the study-site (CHR-TSEVIE).
The wound closes in most cases by itself and adequate wound dressing is sufficient. In case of post-interventional bleeding it may be necessary to apply 1-2 stitches (SERALON 2-0 resp. 3-0). Stitches are usually removed after 7-10 days.

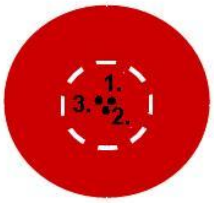

Fig. 2. Punch biopsy, non-ulcerative lesion (e.g. nodule, plaque).
Black dots indicate 3 possible locations for collection of a punch biopsy.

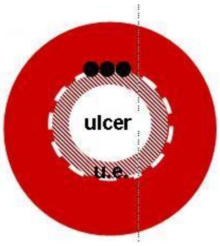

Fig. 3. Punch biopsy, ulcerative lesion without undermined edge.
Black dots indicate 3 possible locations for collection of a punch biopsy.

Surgical tissue samples

Surgically excised tissue specimens are collected at the time of surgery (if conducted for individual patients).
Diagnostic tissue specimens from ulcerative lesions are collected from the edge of the lesion below the end of the undermined edge of the ulcer and must contain necrotic tissue. Surgically excised tissue from pre-ulcerative lesions, such as nodules and plaques, must contain material from the centre of the lesion. (Fig. 4)
A new pair of sterile gloves is used to prepare sterile forceps and sterile scalpel. The excised tissue is spread on sterile gauze. For diagnostic purposes it is mandatory that tissue specimens contain the subcutaneous adipose tissue as Mycobacterium ulcerans is found in that layer. Tissue from non-ulcerative lesions is cut longitudinally and horizontally in four equal segments, each of those must comprise a part from the centre of the lesion. Pieces of a maximum size of 1 cm x 1 cm are then transferred to the respective specimen collection containers containing 700 µl CLS for PCR. For microscopy 1 slide is prepared on study-site (CHR-TSEVIE) and stored in a slide box.

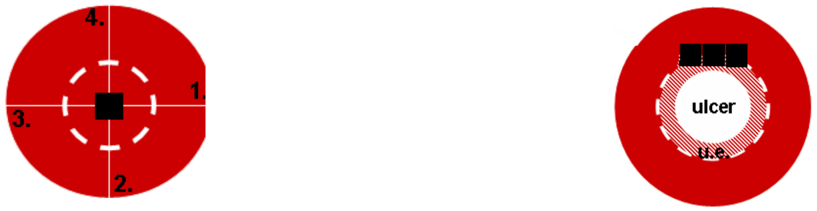

Fig. 4. Diagnostic tissue specimens,
left: pre-ulcerative lesions; right: ulcerative lesion
Black squares indicate the location for collection. u.e.: undermined edge of ulcer.

3. Transport of clinical samples

Diagnostic samples are transported in the respective collection bags including laboratory data entry forms (Form S1) and BU01 forms to INH laboratory by DAHWT.
Specimens for EQA (microscopy) are sent to INH once a week by CHR-Maritime. Before PCR is available at INH, diagnostic samples (swab, FNA or punch biopsy samples) are sent to DITM/LMU on a monthly basis for PCR (FedEx courier service). As soon as PCR equipment is available at INH and local staff has been trained, PCR will be conducted at INH (EQA at DITM/LMU).

4. Storage of diagnostic specimens and immunology samples

Upon arrival at INH laboratories, all diagnostic samples are stored in CLS (700 µl for swabs, punch and tissue samples and 300 µl for FNA) at 2-8 °C until further processing.
According to the three phases of PCR installation at INH laboratory (see 2.1) long term storage of PCR extracts at -20° C is conducted at DITM/LMU for phase 1, at DITM/LMU and INH for phase 2 and at INH only upon launching of phase 3.

Annex

DAHWT- TOOLBOX – GENERAL EQUIPPEMENT FOR DIAGNOSTICS & PATIENT MANAGEMENT					
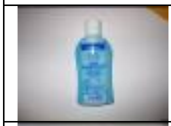	01 flacon	Sterillium	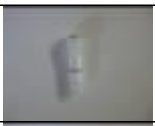	10 units	2 ml screw cap tube with CLS
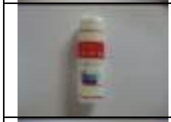	01 flacon	Povidane Iodine 10 %	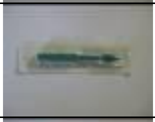	05 units	Biopsy Punch
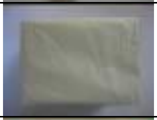	100 units	Compresses 10X10	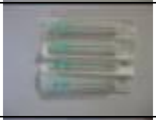	05	Needles
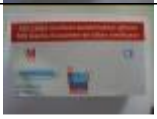	10	Latex gloves, non-stérile	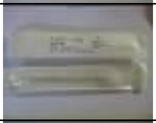	10 unités	Swabs with wooden stick
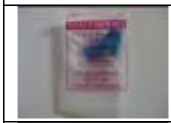	05 units	Scalp Vein Set	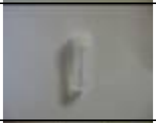	10 unités	Javell water
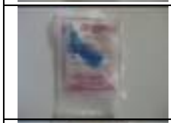	05 units	Robinettes	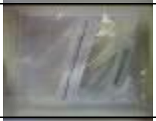	10 x	Plastic bags
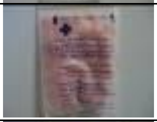	40	IBUPROFEN 400 mg	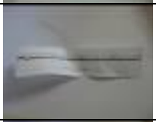	10 units	Measuring tape
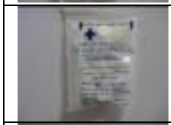	40	Paracetamol 500 mg	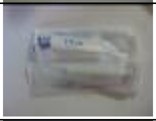	05 units	Syringes, 10 ml
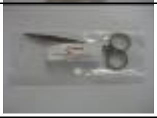	01 unit	Scissors	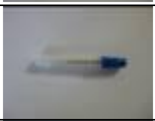	02 unités	Thermometer
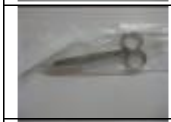	01 unit	Scissors	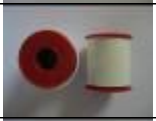	02 unités	Zinc Oxyde Plaster
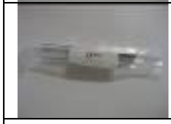	01 unit	forceps	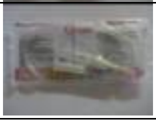	05 unités	Perfusion line
	50 x	Slides for Microscopy		10 x	Biopsy punch, 3 mm
	10 x	2 ml screw cap tubes with 700 µl CLS for punch biopsies			
