# Supplementary material for: Implementation of a National Reference Laboratory for Buruli Ulcer Disease in Togo
Source: PLoS Negl Trop Dis. 2013 Jan 24;7(1):e2011. doi: 10.1371/journal.pntd.0002011 (PMC3554568; doi:10.1371/journal.pntd.0002011)
Supplement: SOP S2 — Microscopic analysis for the detection of acid fast bacilli. (PDF) [file pntd.0002011.s004.pdf]

Standard Operating Procedure (SOP)

Microscopy

Microscopic analysis for the detection of acid fast bacilli

1. General considerations

This document describes the standard operating procedure for microscopic analysis for the detection of acid fast bacilli in clinical samples.

Analysis of specimens for the detection of acid fast bacilli by microscopy comprises 2 steps:

1) Preparation and hot staining of slides
2) Microscopic examination and analysis of slides

Slides are prepared from Swab and FNA specimens (direct smear) in the field or on wards.

2. Preparation for staining of slides

2.1 Reagents

- Ziehl's solution (Carbol Fuchsin, Phenol – commercially available)
- Methylene Blue – solution **III**
- Sulphuric Acid 20 % (H_2SO_4)
- Ethanol 95%
- Distilled water
- Immersion oil

2.2 Materials and instruments

- Slides with frosted edges
- Drying rack
- Gloves (disposable, non sterile)
- Microscope

2.3 Preparation of reagents

<u>Solution I – Ziehl's solution</u>

Filter the Ziehl's solution before use to remove fuchsin crystals or particles!

<u>Solution II – decolourising solution</u>

Dilute 3 ml Sulphuric Acid (20 %) slowly in 97 ml Ethanol (95 %) in a falcon.

▲ ***Never add water to sulphuric acid!***

3. Hot staining of slides

The frosted ends of slides are labelled with the BuruliVac patient ID in the field/on the ward.

Staining procedure is carried out as follows:

- Slides are flooded with filtered Ziehl's solution (**I**) to cover the whole area of the slides.

▲ ***Slides should be properly spaced to avoid cross contamination!***

- The underside of the slide is heated until rising of steam.

  ▲ ***Do not overheat or boil!***

- Allow heated stain to cool down to room temperature for 5 minutes.
- Slides are gently rinsed with tap water
- Decolourising solution (**II)** is added until it completely decolorizes.
- Slides are rinsed gently with water.
- Methylene Blue solution (**III**) is added for 30 seconds
- Slides are rinsed with water.
- Slides are placed in a rack to air dry.

4. Microscopic examination and analysis of slides

4.1 General considerations

Microscopic examination of stained slides is performed using 100-fold magnification with immersion oil. 100 fields should be analyzed. Immersion oil can be stored at room temperature.
In case slides are not analyzable due to wrong preparation in the field or on the ward, a new slide must be collected from the respective patient by the BUD team!

4.2 Performance of microscopic examination and analysis of slides

Slides are examined with 100-fold immersion oil objective in a horizontal manner. Slides positive for acid fast bacilli show red coloured rod-like bacilli. The results can be graded into the following categories:

**Result**	**Grading**
More than 10 AFB / field for at least 20 fields	+++ (positive)
1 – 10 AFB / field	++ (positive)
10 – 99 AFB / 100 fields	+ (positive)
1-9 AFB / 100 fields	marked with exact number / 100 fields (positive)
No AFB in at least 100 fields	- (negative)

Table 1. Microscopy: Result of AFB microscopy, grading system

The results are entered in the BuruliVac "MIC results" form.

5. Quality assurance

The first reading of all slides is conducted at the laboratory of CHR Tsévié including internal quality assurance (IQA). The final result of CHR is determined and entered in the BuruliVac "MIC result – CHR Tsévié" form. Slides and forms are then forwarded via DAHWT office to

the INH laboratory. “MIC result” forms are directly handed over to the datamanager. Slides are re-read at INH for first step external quality assurance (EQA). The final result of INH is determined and entered in the BuruliVac “MIC result – INH, Lomé” form. In case the data manager observes discordant results between CHR and INH, the respective slide must be re-read by the two testers of CHR and INH to confirm the final result for Togo. Next, slides are forwarded to DITM/LMU for external quality assurance.

6. References

WHO (B). Buruli ulcer. Diagnosis of Mycobacterium ulcerans disease. Geneva: WHO, 2001 (Portaels F, Johnson P and M. MW, eds. A manual for health care providers).
