# Supplementary material for: Implementation of a National Reference Laboratory for Buruli Ulcer Disease in Togo
Source: PLoS Negl Trop Dis. 2013 Jan 24;7(1):e2011. doi: 10.1371/journal.pntd.0002011 (PMC3554568; doi:10.1371/journal.pntd.0002011)
Supplement: SOP S3 — Extraction of mycobacterial DNA from clinical specimens. (PDF) [file pntd.0002011.s005.pdf]

# Standard Operating Procedure (SOP)

## DNA extraction

**Extraction of mycobacterial DNA from clinical specimens**

## 1. General considerations

This document describes the standard operating procedure for extraction of mycobacterial DNA from clinical specimens.

DNA extraction is performed in the “DNA extraction laboratory”. This laboratory is exclusively used for this purpose and is free of DNA amplicons.

▲ ***The laboratory is equipped with all necessary lab equipment (i.e. set of pipettes, pipette filter tips, gloves and lab coats) that are exclusively used in this room. Lab coats and gloves must be changed when changing rooms.***

## 2. DNA Extraction

For DNA extraction procedures the Puregene DNA extraction kit (Qiagen, Germany) is used with minor modifications of the manufacturer’s protocol as described below.

### 2.1. Reagents

- Cell Lysis Solution (CLS, Qiagen, Germany)
- DNA Hydration Solution (DNA Hyd, Qiagen)
- Protein Precipitation Solution (PPS, Qiagen)
- Lysozyme (10 mg/ml) (Qiagen)
- Proteinase K (20 mg/ml) (Qiagen)
- Ethanol 70% (e.g. Applichem, Germany)
- 2-Propanol (Isopropanol) (e.g. Applichem)

### 2.2. Materials and instruments

- Lab coats
- Gloves (disposable, non sterile)
- Pipettes (0,1-10 µl, 2-20 µl, 20-100 µl, 100-1000 µl)
- DNase-free Pipette filter tips (10 µl, 20 µl, 100 µl, 1000 µl)
- DNase-free reaction tubes 1,5 ml, 2 ml
- Alarm clock
- Ice bath
- Reaction tube rack
- Table centrifuge
- Thermomixer
- Vortex
- Water resistant pens
- Disinfectant (e.g. Incidin)
- Paper towels, soap, hand disinfectant

## 2.3. Storage and preparation of reagents

▲***All reagents are checked for their date of expiry before use.***

▲***Thaw frozen reagents completely to room temperature before use.***

To minimize contamination risks all reagents are aliquoted in single-use-volumes and stored as described below:

- CLS buffer is used as transport- and storage medium for diagnostic PCR samples. It is aliquoted by 700 µl (300 µl for FNA samples) and stored at room temperature.
- PPS-solution and DNA Hyd are stored at room temperature and aliquoted according to the amount of samples subjected to DNA extraction.
- Isopropanol is aliquoted to 700 µl and stored at room temperature in the dark.
- Ethanol (70%) is stored at room temperature in the dark and filled from the bottle to a 50 ml falcon tube before use (if ethanol needs to be diluted from 100% to 70%: 35 ml of Ethanol 100% is added to 15 ml distilled water directly before use). Any remaining ethanol in the 50 ml falcon tube is discarded directly after use.
- Lysozyme 10 mg/ml is aliquoted by 100 µl, aliquots are stored at –20°.
- Proteinase K 20 mg/ml is aliquoted by 100 µl, aliquots are stored at –20°.

## 2.4. Performance of DNA Extraction

Using the Qiagen Puregene DNA extraction kit, DNA may be extracted from the following samples according to the below mentioned protocols:

A) Surgically excised tissue material (maximum size 1 x 1 cm), 3 mm punch biopsies
B) Fine needle aspirates
C) Swab samples

DNA extracts are ready for subjection to PCR.

With every new extraction of diagnostic samples, one negative extraction control* should be processed.

### *2.4.1 Prearrangements*

After arrival of samples at the laboratory, specimens for PCR analysis are heat-inactivated by incubation at 95°C in the thermomixer for 15 minutes.

▲***Specimens are not infectious anymore but nevertheless should be treated as "possibly infectious" agents and handled with care!***

The volume of CLS buffer surrounding the samples is checked. All tubes containing less CLS should be adjusted to the required amounts of 700 µl (swab, punch and tissue samples) or 300 µl (FNA samples) by adding CLS.

▲***The samples are labelled with patient's ID to ensure the correct allocation of patients and samples.***

*Before starting the extraction procedure, prepare one empty reaction tube as negative extraction control: Add 700 µl CLS buffer. The extraction control will be treated the same way as the samples, but must not contain any diagnostic sample.

### 2.4.2 *DNA extraction from surgically excised tissue, punch biopsy and FNA samples*

**Cell lysis**

1. Add **10 µl Proteinase K [20 mg/ml]** to each sample and incubate at 55°C in the thermomixer shaking at moderate speed overnight until complete lysis.
2. Inactivate Proteinase K by incubation at 80°C for 20 minutes in the thermomixer.
3. Cool down samples to room temperature, add **15 µl Lysozyme [10 mg/ml]** and incubate at 37°C in the thermomixer at moderate speed for 1 hour.

**Protein Precipitation**

1. Place samples in an ice bath for 5 minutes.
2. Add 230 µl Protein Precipitation Solution (PPS).
3. Vortex vigorously at high speed for 20 seconds.
4. Place samples in an ice bath for 5 minutes.
5. Centrifuge at 13.000 x g for 5 minutes. The precipitated proteins will form a tight pellet. If protein pellets are not tight, repeat step 3-5.
   During centrifugation prepare the respective number of new 2 ml reaction tubes containing 700 µl Isopropanol.

**DNA Precipitation**

1. Pour supernatant containing DNA (leaving behind the precipitated protein pellet) into a clean 2 ml reaction tube containing 700µl Isopropanol.
2. Mix by inverting gently 10 times.
3. Centrifuge at 13.000 x g for 5 minutes.
4. Pour off the supernatant. Add 700 µl of Ethanol 70% and invert the tubes to wash DNA pellets.
5. Centrifuge at 13.000 x g for 5 minutes. Carefully pour off ethanol. The pellet may be loose so pour slowly and watch the pellets.
6. Invert and drain tubes on a clean absorbent paper towel (each tube at a different spot) and allow to air dry or in the thermomixer at 65°C until tubes are completely dry (20 min.). Ethanol must be completely evaporated as this may inhibit PCR.

**DNA Hydration**

1. Add **200µl** DNA Hyd (**50 µl** for **FNA** samples).
2. Rehydrate DNA by carefully pipetting up- and down about 20 times.
3. Incubate in the thermomixer for 30 minutes at 65°C.

### *2.4.3 DNA extraction from swab samples*

**Cell lysis**

4. 1. Add **15 µl Lysozyme [10 mg/ml]** to each sample and incubate at 37°C in the thermomixer shaking at moderate speed for 1 hour.
2. Add **10 µl Proteinase K [20 mg/ml]** to samples and incubate at 55°C in the thermomixer at moderate speed for 4 hours.
3. Proteinase K is inactivated at 80°C for 15 minutes in the thermomixer.

**Protein Precipitation**

6. Place samples in an ice bath for 5 minutes.
7. Add 230 µl Protein Precipitation Solution (PPS).
8. Vortex vigorously at high speed for 20 seconds.
9. Place samples in an ice bath for 5 minutes.
10. Centrifuge at 13.000 x g for 5 minutes. The precipitated proteins will form a tight pellet. If protein pellets are not tight, repeat step 3-5.
    During centrifugation prepare the respective number of new 2 ml reaction tubes containing 700 µl Isopropanol.

**DNA Precipitation**

7. Pour supernatant containing DNA (leaving behind the precipitated protein pellet) into a clean 2 ml reaction tube containing 700µl Isopropanol.
8. Mix by inverting gently 10 times.
9. Centrifuge at 13.000 x g for 5 minutes.
10. Pour off the supernatant. Add 700 µl of Ethanol 70% and invert the tubes to wash DNA pellets.
11. Centrifuge at 13.000 x g for 5 minutes. Carefully pour off ethanol. The pellet may be loose so pour slowly and watch the pellets.
12. Invert and drain tubes on a clean absorbent paper towel (each tube at a different spot) and allow to air dry or in the thermomixer at 65°C until tubes are completely dry (20 min.). Ethanol must be completely evaporated as this may inhibit PCR.

**DNA Hydration**

4. Add **200µl** DNA Hyd.
5. Rehydrate DNA by carefully pipetting up- and down about 20 times.
6. Incubate in the thermomixer for 30 minutes at 65°C.

## 3. Storage of DNA Extracts

DNA extracts can be stored at 4°C until further processing for up to 1 week. For long term storage DNA extracts are conserved at –20°C.

Avoid repeated freezing and thawing of extracts as this may lead to degradation of DNA.
