# Supplementary material for: Implementation of a National Reference Laboratory for Buruli Ulcer Disease in Togo
Source: PLoS Negl Trop Dis. 2013 Jan 24;7(1):e2011. doi: 10.1371/journal.pntd.0002011 (PMC3554568; doi:10.1371/journal.pntd.0002011)
Supplement: SOP S4 — IS2404-DRB-PCR for detection of M. ulcerans DNA. (PDF) [file pntd.0002011.s006.pdf]

Standard Operating Procedure (SOP)

IS2404-DRB-PCR

_IS2404_-DRB-PCR and agarose gel-electrophoresis for detection of _M. ulcerans_ DNA

1. General considerations

This document describes the standard operating procedure for *IS2404*-DRB-PCR and agarose gel-electrophoresis for detection of *M. ulcerans* DNA from clinical samples.

Preparation of the **d**ry **r**eagent **b**ased (**DRB**) PCR must be performed in the "Master Mix laboratory". This laboratory is exclusively used for this purpose and is free of DNA amplicons.
The laboratory must be equipped with lab coats, gloves and all necessary laboratory items exclusively used in this room.

DRB-PCR consists of 2 major steps:

1. DRB-PCR
2. Agarose gel-electrophoresis

2. DRB-PCR

2.1. Reagents

- PuReTaq Ready-To-Go PCR Beads (GE Healthcare, Munich, Germany)
- Lyophilised primers MU5 and MU6 (e.g. TibMolBiol, Berlin, Germany)
- Purified distilled water, DNAse free (e.g. Roth, Karlsruhe, Germany)

2.1.1. Storage and preparation of reagents

PuReTaq Ready-To-Go PCR Beads are stored at room temperature and checked for their date of expiry before use.
Lyophilised primers in 0.2 ml PCR reaction tubes are stored at room temperature.
To minimize risks of contamination, distilled water is pre-filled at 1 ml aliquots and stored at 4°C. Each aliquot is used for set-up of one DRB-PCR and discarded.

2.2. Materials and instruments

- Pipettes (0,1-10 µl, 2-20 µl, 20-100 µl, 100-1000 µl)
- DNase-free Pipette filter tips (10 µl, 20 µl, 100 µl, 1000 µl)
- DNase-free reaction tubes 1,5 ml, 2 ml
- DNase-free reaction tubes 0,2 ml containing 10µM lyophilised primers MU5 and MU6
- Reaction tube rack
- Gloves (disposable, non sterile)
- Water resistant pen
- Mini table centrifuge
- Vortex
- Thermocycler

2.3. Performance of DRB-PCR

Preparation of master mix

▲ ***The following steps are performed in the "Master Mix laboratory"!***

- PuReTaq Ready-To-Go-PCR-beads are added to reaction tubes (containing pre-lyophilised primers MU5 + MU6) one each.
- 22,5 µl distilled water are added.
- Before adding DNA from extracts or controls beads must be dissolved completely.

▲ ***Ready-To-Go-PCR beads contain PCR buffer, $MgCl_2$ and dNTPs.***

Addition of template

▲ ***PCR templates must be added in "DNA extraction laboratory"!***

Diagnostic samples
Every diagnostic sample (DNA extract) is tested undiluted and in 1:10 dilution.
2.5 µl DNA extract or dilution is added to the respective tubes.

Inhibition controls
To prevent false negative results due to inhibition processes inhibition controls (undiluted and diluted) are processed in parallel to all diagnostic samples.
For inhibition control reactions 1.25 µl of diagnostic (unknown) DNA or dilution and 1.25 µl positive control DNA is added to the respective tubes.

Positive and negative controls
Apart from the diagnostic samples and inhibition controls following controls are processed:

Type of control	Purpose	Content of reaction
**negative extraction control**	Negative control of the extraction process to exclude contamination during extraction	2.5 µl of the negative extraction control
**negative (no template) PCR control**	Negative control of PCR, to exclude contamination of PCR reagents	2.5 µl distilled water without any DNA
**positive PCR control***	Positive control (run control) of PCR to determine specific amplification	2.5 µl positive DNA

*As positive control either an already positively tested patient DNA extract may be used, or confirmed culture extracts.

Amplification

After preparation of reaction mixtures all samples are transferred to the thermocycler. Amplification should be carried out according to the following protocol.
Duration of PCR: 1:30 h

Steps	Temperature	Duration	Number of cycles
**Initial Denaturation**	95°C	10 minutes	1
**Denauration**	95°C	10 seconds	40
**Annealing of primers**	58°C	10 seconds	
**Extention**	72°C	30 seconds	
**Final extention**	72°C	10 minutes	1
**Hold**	15°C	∞	

3. Agarose gel-electrophoresis

3.1. General considerations

Agarose gel-electrophoresis is performed in the “Agarose gel-electrophoresis laboratory”.

▲ ***This laboratory is exclusively used for gel-electrophoresis and must not be used for DNA extraction, master mix preparation or DRB-PCR!***

The laboratory must be equipped with lab coats, gloves and all necessary laboratory items exclusively used in this room. No change of material/equipment between this room and other rooms is allowed.

3.2. Reagents

- Agarose low EEO (standard agarose, e.g. Applichem)
- 10x TBE buffer (e.g. Roth)
- GelRed™ (Biotium, Hayward, CA)
- 10x loading dye Bluejuice (e.g. Invitrogen, Darmstadt, Germany)*
- DNA ladder, 100 bp (e.g. Invitrogen)

*Loading dye can be alternatively prepared by mixing glycerol (50 %) with Bromophenolblue (0.25 %) and distilled water (49.75%).

3.2.1 Storage and preparation of reagents

All reagents are checked for their date of expiry before use.
Concentrated DNA ladder is prepared to a working solution by adding 10 µl of concentrated DNA ladder and 10 µl of 10 x Bluejuice to 80 µl of distilled water.
The concentrated DNA ladder is stored at -20°C.
TBE-buffer and agarose can be stored at room temperature. DNA ladder in working solution and Bluejuice must be stored in the fridge (4-8°C).

3.3. Materials and instruments

- Lab coat
- Gloves (disposable, nitrile)
- Pipettes (0,1-10 µl, 2-20 µl)
- DNase-free Pipette tips (10 µl, 20 µl)
- DNase-free reaction tubes 1,5 ml,
- Reaction tube rack
- Erlenmeyer flask, plastic
- Water resistant pen
- UV-Light Photo documentation System
- Electorophoresis chamber & Power supply
- Microwave oven

3.4. Performance of agarose gel-electrophoresis

The working concentration for the TBE buffer is 0.5 fold, thus concentrated 10x TBE buffer is diluted 1:20 fold (50 ml of 10x TBE buffer are added to 950 ml distilled and deionised water).
For preparation of a 1.5 % agarose gel 1.5 g agarose and 100 ml of 0.5x TBE Buffer are heated in a plastic Erlenmeyer flask for 3 minutes at 600 W in a microwave oven. If the agarose is not fully melted the cooking time needs be increased.

▲ ***Bubbles indicate the right heat!***

Following heating the fluid needs beeing cooled down to approximately 50°C. Therefore, the flask may be put under cool running water. Shaking the flask ensures cooling of the fluid uniformly.
After cooling, 10 µl of GelRed are added to the fluid. To uniformly disperse GelRed in the flask, it is shaken.
The electrophoresis tray is sealed and the fluid is poured without producing bubbles into agarose gel-electrophoresis tray. Bubbles are removed carefully by using a pipette tip. Then the spacer comb(s) is/are placed at its correct location at the agarose gel-electrophoresis tray.
The agarose gel is then allowed to cool down to room temperature. Then spacer comb(s) is (are) removed carefully.
The congealed gel is put into the agarose gel-electrophoresis chamber, which should already be filled with 0.5x TBE buffer up to the filling line.
To load the gel, 15 µl of each DRB-PCR amplicon is mixed with 3 µl of 10x Bluejuice. The mix is entered completely and carefully into the respective sample slot. 10 µl of diluted 100bp DNA ladder is loaded onto one sample slot per line.
Voltage is applied and electrophoresis is carried out according to the following table.

Size of gel	Voltage	No. of spacer combs	Time of gel-electrophoresis
12 x 12 cm	100 V	2	55 minutes

3.5. Analysis and interpretation of agarose gel-electrophoresis

After completion of agarose gel-electrophoresis amplicons can be visualized using UV light (302 nm) in the gel documentation unit.
A positive reaction produces a band of **492 bp in length**; a negative reaction produces no band.
Results of diagnostic samples are interpreted only according to the results of corresponding results from inhibition controls. The following table indicates all possible results.

Result of sample	Result of inhibition control	Interpretation of results
Negative	Positive	Sample negative
Positive	Positive	Sample positive
Negative	Negative	Sample inibite*
Positive	Negative	Sample positive

*Repeat testing of sample(s) in higher dilution

Protocol: DRB-PCR for molecular confirmation of suspected BUD patients

Date……....……...…..

No.	Patient´s samples	Dilution			No.	Inhibition control to corresponding patients' samples	No.	Positive- and negative controls
**1**		10	E		**11**	1	**21**	Negative extraction control
**2**		10	E		**12**	2	**22**	Positive PCR control
**3**		10	E		**13**	3	**23**	Negative PCR control
**4**		10	E		**14**	4	**24**	
**5**		10	E		**15**	5	**25**	
**6**		10	E		**16**	6		
**7**		10	E		**17**	7		
**8**		10	E		**18**	8		
**9**		10	E		**19**	9		
**10**		10	E		**20**	10		

Preparation of DRB-PCR reaction mix

- Add 1 PuRe Taq ™Ready-To-Go PCR bead to each tube containing lyophilized primers (#MU5 + #MU6, 1,25 µl each, 10 µM)
- Add **22.5 µl water** to
  - **each patient´s sample tube** (No. 1-10)
  - **each inhibition control tube** (No. 11-20)
  - **the negative extraction control tube** (No. 21)
  - **the positive PCR control tube** (No. 22)
- Add **25 µl water** to **negative PCR control tube** (No. 23)
- Add **2.5 µl DNA extract** to the corresponding **patient´s sample tube** (No. 1-10)
- Add **1.25 µl of patient DNA extract** and **1.25 µl of positive control** DNA to the corresponding inhibition control tubes (No. 11-20)
- Prepare **premix for inihibition controls**:
- Add **2.5 µl extraction control "extract"** to the corresponding tube (No. 21)
- Add **2.5 µl positive PCR control** to the corresponding tube (No. 22)

Thermal cycling	
**95°C, 10 min**	1 cycle
**95°C, 10 sec**<br>**58°C, 10 sec**<br>**72 °C, 30 sec**	40 cycles
**72°C, 10 min**	1 cycle
**15°C, hold**	~

Agarose gel-electrophoresis

Heat in a microwave (until dissolving):

- **1,5 g agarose**
- **100 ml 0.5x TBE-Buffer**

let cool down to 50°C

- add **10 µl GelRed**

Pour liquid gel in gelelectrophoresis tray

- Add **spacer combs**

let cool down to RT

Put gel into gelelectrophoresis chamber

Add **0.5x TBE-Buffer** (up to fill line)

Add **3 µl BlueJuice (loading dye) to each PCR tube**

Pipette 12-15µl into **gel slots**

Pipette 7-10 µl 100 bp-DNA ladder working solution into one gel slot per line

Apply voltage (100 V)

Run electrophoresis for 55 minutes

-UV transillumination and photo documentation-

Results (gel-picture)

Positive patients	Negative patients	Inhibited patients

4. External quality assurance

External quality assurance is carried out at DITM/LMU laboratories. In accordance with different phases of the PCR installation at INH, specimens are initially tested at INH and DITM/LMU in parallel, see SOP S1 “Collection, transport and storage of diagnostic specimens”. If results obtained at INH are throughout concordant with results of DITM/LMU the final phase starts. Then, aliquots of all extracts (25 µl for swabs and punch biopsy extracts, 10 µl for FNA extracts) are sent quarterly to DITM/LMU by means of DHL courier service for external quality assurance.

5. References

Stinear, T., B. C. Ross, J. K. Davies, L. Marino, R. M. Robins-Browne, F. Oppedisano, A. Sievers, and P. D. Johnson. **1999**. Identification and characterization of IS2404 and IS2606: two distinct repeated sequences for detection of Mycobacterium ulcerans by PCR. J Clin Microbiol 37:1018-23.

Siegmund V, Adjei O, Racz P, Berberich C, Klutse E, van Vloten F, Kruppa T, Fleischer B, Bretzel G, **2005**. Dry-reagent-based PCR as a novel tool for laboratory confirmation of clinically diagnosed Mycobacterium ulcerans-associated disease in areas in the tropics where M. ulcerans is endemic. J Clin Microbiol 43: 271-6.

Siegmund V, Adjei O, Nitschke J, Thompson W, Klutse E, Herbinger KH, Thompson R, van Vloten F, Racz P, Fleischer B, Loescher T, Bretzel G, **2007**. Dry reagent-based polymerase chain reaction compared with other laboratory methods available for the diagnosis of Buruli ulcer disease. Clin Infect Dis 45: 68-75.

Bretzel G, Siegmund V, Nitschke J, Herbinger KH, Thompson W, Klutse E, Crofts K, Massavon W, Etuaful S, Thompson R, Asamoah-Opare K, Racz P, Vloten F, van Berberich C, Kruppa T, Ampadu E, Fleischer B, Adjei O, **2007**. A stepwise approach to the laboratory diagnosis of Buruli ulcer disease. Trop Med Int Health 12: 89-96.
